# Transcriptional changes in the peripheral blood leukocytes from Brangus cattle before and after tick challenge with *Rhipicephalus australis*

**DOI:** 10.1186/s12864-022-08686-3

**Published:** 2022-06-20

**Authors:** Emily F. Mantilla Valdivieso, Elizabeth M. Ross, Ali Raza, Muhammad Noman Naseem, Muhammad Kamran, Ben J. Hayes, Nicholas N. Jonsson, Peter James, Ala E. Tabor

**Affiliations:** 1 The University of Queensland, Queensland Alliance for Agriculture and Food Innovation, Centre for Animal Science, St Lucia, Queensland 4072 Australia; 2 University of Glasgow, Institute of Biodiversity Animal Health and Comparative Medicine, Glasgow, G61 1QH UK; 3 The University of Queensland, School of Chemistry and Molecular Biosciences, St Lucia, Queensland 4072 Australia

**Keywords:** RNA-seq, Cattle, Biomarkers, Cattle tick, Host resistance, Bovine, Brangus

## Abstract

**Background:**

Disease emergence and production loss caused by cattle tick infestations have focused attention on genetic selection strategies to breed beef cattle with increased tick resistance. However, the mechanisms behind host responses to tick infestation have not been fully characterised. Hence, this study examined gene expression profiles of peripheral blood leukocytes from tick-naive Brangus steers (*Bos taurus x Bos indicus*) at 0, 3, and 12 weeks following artificial tick challenge experiments with *Rhipicephalus australis* larvae. The aim of the study was to investigate the effect of tick infestation on host leukocyte response to explore genes associated with the expression of high and low host resistance to ticks.

**Results:**

Animals with high (HR, *n* = 5) and low (LR, *n* = 5) host resistance were identified after repeated tick challenge. A total of 3644 unique differentially expressed genes (FDR < 0.05) were identified in the comparison of tick-exposed (both HR and LR) and tick-naive steers for the 3-week and 12-week infestation period. Enrichment analyses showed genes were involved in leukocyte chemotaxis, coagulation, and inflammatory response. The IL-17 signalling, and cytokine-cytokine interactions pathways appeared to be relevant in protection and immunopathology to tick challenge. Comparison of HR and LR phenotypes at timepoints of weeks 0, 3, and 12 showed there were 69, 8, and 4 differentially expressed genes, respectively. Most of these genes were related to immune, tissue remodelling, and angiogenesis functions, suggesting this is relevant in the development of resistance or susceptibility to tick challenge.

**Conclusions:**

This study showed the effect of tick infestation on Brangus cattle with variable phenotypes of host resistance to *R. australis* ticks*.* Steers responded to infestation by expressing leukocyte genes related to chemotaxis, cytokine secretion, and inflammatory response. The altered expression of genes from the bovine MHC complex in highly resistant animals at pre- and post- infestation stages also supports the relevance of this genomic region for disease resilience. Overall, this study offers a resource of leukocyte gene expression data on matched tick-naive and tick-infested steers relevant for the improvement of tick resistance in composite cattle.

**Supplementary Information:**

The online version contains supplementary material available at 10.1186/s12864-022-08686-3.

## Background

*Rhipicephalus microplus* is a tick species complex of hematophagous arthropods that parasitise cattle in tropical and subtropical countries, of which the representative species found in Australia is *Rhipicephalus australis* [1, 2]. Heavy tick infestation and the associated risk of transmission of tick-borne pathogens have detrimental consequences for animal health, welfare, and production in the cattle industry. Global economic losses due to cattle ticks are estimated to be US$22–30 billion annually [3–5]. Strategies to control the spread of cattle ticks include treatment with acaricides, pasture spelling, the use of tick-resistant cattle, or a combination of all of these [6]. However, treating animals with synthetic acaricides raises concerns about potential residual effects in meat and milk for human consumption, as well as the challenge of the development of chemical resistance [7, 8]. Genetic selection to improve host resistance to ectoparasites remains the most sustainable approach to minimise disease burden and enhance animal welfare in the cattle industry [5, 9, 10].

Female cattle ticks have an average parasitic phase of ~ 21 days on the host. During this period, the ticks secrete a diverse range of bioactive molecules to modulate host responses in order to achieve feeding success and survival [11]. This complex range of secreted protein and non-protein tick salivary molecules has been reviewed elsewhere [12]. Some of the host processes that are known to be disrupted by the hematophagy of cattle ticks include blood coagulation, cytokine secretion, cell adhesion, as well as leukocyte recruitment to the feeding site. Conversely, host resistance in cattle manifests by preventing attachment of tick larvae, which results in early larval death, as well as limiting hematophagy, thus reducing the reproductive success of the female adult ticks [11].

Host resistance is a moderately heritable trait which can range from high (tick-resistant) to low (tick-susceptible) as determined by the number of engorging adult ticks (4.5-8 mm) following a natural or artificial larval infestation [13, 14]. *Bos indicus* cattle generally have very high tick resistance, whereas *Bos taurus* cattle are mostly susceptible [14]. However, stable composite breeds (*B. indicus* x *B. taurus*) range widely from low to high host resistance as reported in Santa-Gertrudis [15], Bradford [16] and Brangus [17]. Targeting tick resistance in breeding programs is desirable, but measuring the phenotype requires collection of tick counts or scores on an individual animal basis, which is costly, laborious, and requires standardisation of exposure [5, 9]. Hence, knowledge of the genes controlling this trait will contribute towards the strategic development of biomarker-assisted selection methods for tick resistant cattle.

Gene expression and quantitative trait loci (QTL) studies have previously shown that host resistance to the cattle tick is controlled by multiple genes [16, 18–25]. Additionally, the complex interplay of innate, adaptive and mal-adaptive responses elicited during tick infestation have been shown to contribute to the expression of divergent phenotypes [15, 26–35]. As a result, identification of genes that can be used as universal biomarkers of tick resistance is particularly challenging. Transcriptomic profiling of resistant versus susceptible cattle has been well characterised at the skin level [16, 18, 19, 27, 36, 37], the primary site of tick attachment and feeding, whereas immunologically relevant tissues are still far less explored [21, 26, 31, 38]. Since blood collection is a less invasive procedure than skin biopsy collection for biomarker research, this study hypothesized that it is possible to use peripheral blood leukocytes to elucidate differentially expressed genes that contribute to divergence in host resistance to cattle ticks. Therefore, the aim of this study was to apply RNA-sequencing in the leukocytes of Brangus cattle (*B. indicus* x *B taurus*) collected before and after repeated artificial challenge with *R. australis* tick larvae to identify genes associated with 1) host response to tick infestation; and 2) divergent phenotypes of host resistance.

## Results

### Host resistance phenotyping

**Fig. 1:**
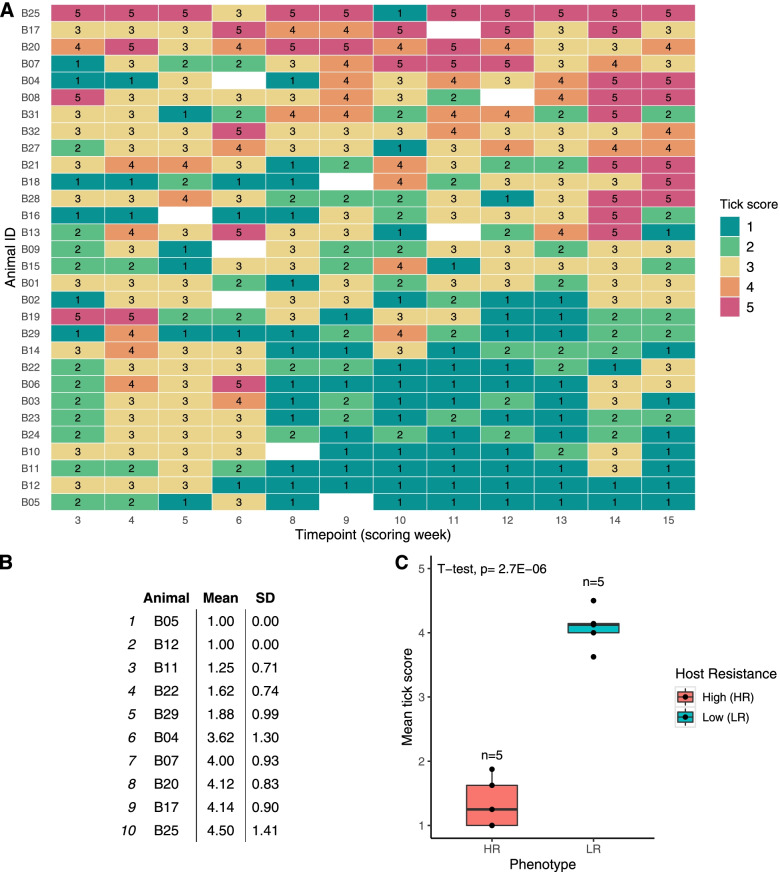
Tick scoring for host resistance phenotyping of Brangus steers. **A** Heatmap representation of the tick scoring data collected from 30 Brangus steers across twelve timepoints showing clustering of the most resistant (bottom) and least resistant (top). Tick scores represent the estimated number of adult ticks that develop 21 days after a single infestation with *R.australis* larvae, where 1 = 0–50 ticks, 2 = 50–100 ticks, 3 = 100–200 ticks, 4 = 200–300 ticks, 5 = more than 300 ticks, blank = not scored. The vertical colour bar indicates the value that each colour represents in the heatmap from low (green) to high (red). **B** The mean tick score (MTS) ± standard deviation (SD) calculated across week 8–15 for 10 animals selected as divergent in host resistant phenotype. **C** Boxplots showing the comparison of MTS (week 8–15) values between the high (HR, *n* = 5) and low (LR, *n* = 5) host resistance phenotypes

Thirty tick-naive Brangus steers were artificially infested with *R. australis* larvae for 12 consecutive weeks and host resistance phenotype was determined by weekly measurement of tick burden with scores (Fig. 1A). The scores were less variable between week 8 and week 15, and this period was considered to represent stabilisation of the phenotype, hence, the mean tick score (MTS) was used to rank animals. A summary of the mean tick scores for 10 animals selected as divergent in host resistance phenotype is given in Fig. 1B. The highly resistant steers (HR, *n* = 5) and least resistant steers (LR, *n* = 5) had a group MTS of 1.35 ± 0.49 and 4.08 ± 1.08, respectively, and the difference was significant (*p* < 0.001) (Fig. 1C). The scoring data table for all 30 Brangus steers is provided in Additional File 1.

### Genomic estimates of *Bos indicus* content and relationship with mean tick score

Genomic estimates of *B. indicus* content (BIC) were used to investigate if there was an association between low tick burdens and high indicine content in this Brangus population. The BIC values obtained for 29 steers ranged from 25 to 49% with a median value of 40% (Additional File 2). The inverse correlation between MTS and BIC values was not significant (*r* = − 0.17, *p* = 0.37), as shown in the figure provided in Additional File 2.

### RNA-sequencing statistics summary

Leukocyte samples from the HR and LR group at three timepoints (T0, T3, and T12) of the infestation trial were included in this RNA-Seq study (see Section “Methods” for further information). Following the removal of two RNA samples from the HR-T0 group, there were 28 sequenced libraries which generated, on average, 36,170,898 raw reads (100 bp single-end) per sample. Of these, 0.3% of the reads were discarded after adapter trimming and read quality control and 93.4% of the remaining sequences were uniquely mapped to the reference *B. taurus* genome ARS-UCD1.2. The average library size was 23,460,531 reads assigned as counts to 28,786 annotated genes in the reference genome. The RNA-seq mapping statistics per sample are shown in Additional File 3.

### Differentially expressed genes associated with host response to tick infestation

To study the effect of tick infestation on leukocyte gene expression, comparisons were performed between timepoint sample sets T3 (*n* = 10) vs. T0 (*n* = 8), and T12 (*n* = 10) vs. T0.

The total number of expressed genes tested by *edgeR* was 13,984 in T3-vs-T0, and 13,865 in T12-vs-T0. Exploratory analysis with multidimensional scaling (MDS) plot showed sample clustering in the first dimension according to timepoint as expected, whereas the secondary dimension separated samples according to host resistance phenotype. This was more clearly evident in T0 than in the T3 and T12 sample sets (see Additional File 4).

**Fig. 2:**
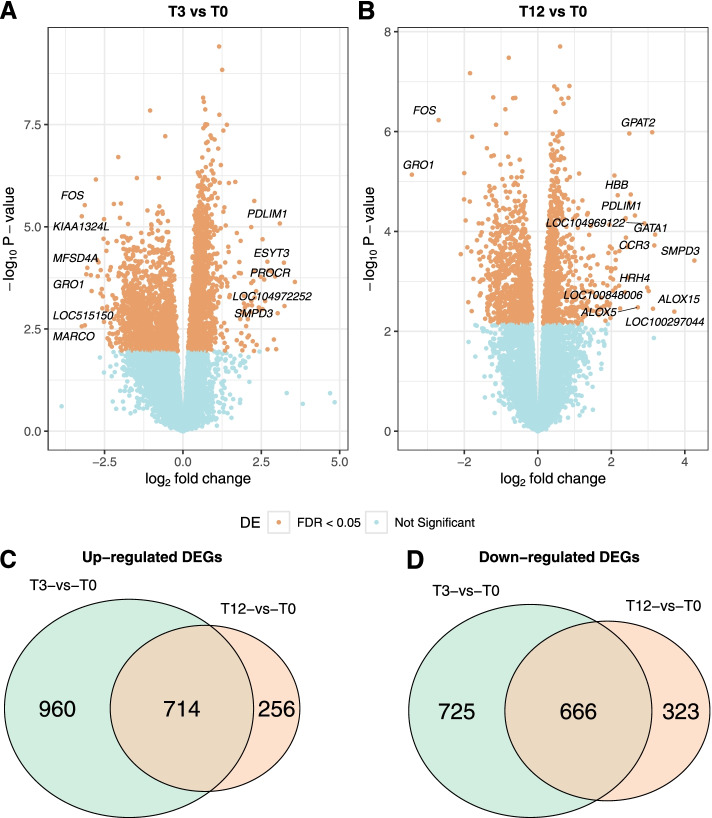
Differentially expressed genes (DEGs) in the leukocytes of tick-infested compared to tick-naive Brangus steers. **A** Volcano plot of DEGs from T3-vs-T0 comparison (3-week tick-exposed vs. tick-naive). **B** Volcano plot of DEGs from T12-vs-T0 comparison (12-week tick-exposed vs. tick-naive). The x-axis represents fold change and y-axis represents statistical significance. Symbols are shown for genes with fold change |logFC| > 2.5 at a significance threshold FDR < 0.05. **C** Venn diagram of upregulated DEGs between comparisons. **D** Venn diagram of downregulated between comparisons

Differential expression analysis in T3-vs-T0 identified 3065 significant DEGs (FDR < 0.05), of which 1674 were upregulated and 1391 were downregulated genes (Fig. 2A; Additional File 5). In the ranked gene list according to expression fold change, the top upregulated (logFC> 3) DEGs were *PROCR*, *LOC104972252*, *ESYT3*, *PDLIM1*, *SPMD3*, *ALOX15*, whereas the top downregulated (logFC< − 3) DEGs were *MARCO*, *KIAA1324L*, *FOS*, *LOC515150*, *GRO1*, *MFSD4A* (Table 1).

**Table 1:** Top significant DEGs from the comparison between 3-week tick-exposed and tick-naive steers

Symbol	Entrez ID	Name	log_**2**_FC^**a**^	p-val	FDR^**b**^
*PROCR*	282,005	protein C receptor	3.57	2.23E-04	4.34E-03
*LOC104972252*	104,972,252	uncharacterized LOC104972252	3.24	8.69E-04	9.71E-03
*ESYT3*	530,157	extended synaptotagmin 3	3.22	7.56E-05	2.39E-03
*PDLIM1*	614,675	PDZ and LIM domain 1	3.09	8.31E-06	7.37E-04
*SMPD3*	514,201	sphingomyelin phosphodiesterase 3	3.02	1.30E-03	1.22E-02
*ALOX15*	282,139	arachidonate 15-lipoxygenase	2.97	9.88E-03	4.67E-02
*MFSD4A*	515,128	major facilitator superfamily domain containing 4A	−3.05	1.01E-04	2.83E-03
*GRO1*	281,212	chemokine (C-X-C motif) ligand 1 (melanoma growth stimulating activity, alpha)	−3.08	1.45E-04	3.51E-03
*LOC515150*	515,150	apolipoprotein R	−3.13	2.57E-03	1.93E-02
*FOS*	280,795	Fos proto-oncogene, AP-1 transcription factor subunit	−3.13	2.96E-06	4.89E-04
*KIAA1324L*	518,313	KIAA1324 like	−3.23	5.54E-06	6.26E-04
*MARCO*	505,632	macrophage receptor with collagenous structure	−3.23	2.73E-03	2.00E-02

^a^ log_2_FC represents the fold change in gene expression between 3-week tick-exposed (T3 sample set) and tick-naive steers (T0 sample set). Table displays genes above threshold |logFC| > 2.5

^b^ FDR represents the false discovery rate (Benjamini-Hochberg multiple test correction of the *p*-value). Table displays genes below threshold FDR < 0.05

The T12-vs-T0 analysis identified 1959 significant DEGs (FDR < 0.05), of which 989 were upregulated and 970 were downregulated genes (Fig. 2B; Additional File 6). In the ranked gene list according to expression fold change, the most upregulated DEGs (logFC> 2.5) were *SPMD3*, *ALOX15*, *GATA*, *CCR3*, *LOC100297044*, *GPAT2*, *LOC100848006*, *HRH4*, *LOC104969122*, *ALOX5*, *PDLIM1*, *HBB*, whereas the most downregulated DEGs (logFC<− 2.5) were *FOS*, *GRO1* (Table 2).

**Table 2:** Top significant DEGs from the comparison between 12-week tick-exposed and tick-naive steers

Symbol	Entrez ID	Name	Log_2_FC^a^	p-val	FDR^b^
*SMPD3*	514,201	sphingomyelin phosphodiesterase 3	4.26	3.83E-04	9.50E-03
*ALOX15*	282,139	arachidonate 15-lipoxygenase	3.71	4.04E-03	3.59E-02
*GATA1*	516,066	GATA binding protein 1	3.19	1.16E-04	5.38E-03
*CCR3*	408,018	C-C motif chemokine receptor 3	3.17	1.91E-04	6.90E-03
*LOC100297044*	100,297,044	C-C motif chemokine 14	3.13	3.53E-03	3.32E-02
*GPAT2*	526,739	glycerol-3-phosphate acyltransferase 2, mitochondrial	3.11	1.03E-06	6.95E-04
*LOC100848006*	100,848,006	uncharacterized LOC100848006	3.02	1.56E-03	2.12E-02
*HRH4*	783,354	histamine receptor H4	2.98	1.34E-03	1.93E-02
*LOC104969122*	104,969,122	uncharacterized LOC104969122	2.90	6.90E-05	4.32E-03
*ALOX5*	404,074	arachidonate 5-lipoxygenase	2.72	3.31E-03	3.21E-02
*PDLIM1*	614,675	PDZ and LIM domain 1	2.64	4.80E-05	3.64E-03
*HBB*	280,813	hemoglobin, beta	2.53	1.82E-05	2.60E-03
*BMPR1B*	407,128	bone morphogenetic protein receptor type 1B	2.50	2.66E-05	2.87E-03
*MUC13*	100,295,610	mucin 13, cell surface associated	2.49	1.09E-06	6.95E-04
*FOS*	280,795	Fos proto-oncogene, AP-1 transcription factor subunit	−2.70	5.90E-07	5.46E-04
*GRO1*	281,212	chemokine (C-X-C motif) ligand 1 (melanoma growth stimulating activity, alpha)	−3.43	7.30E-06	1.71E-03

^a^ log_2_FC represents the fold change in gene expression between 12-week tick-exposed (T12 sample set) and tick-naïve steers (T0 sample set). Table displays genes above threshold |logFC| > 2.5

^b^ FDR represents the false discovery rate (Benjamini-Hochberg multiple test correction of the *p*-value). Table displays genes below threshold FDR < 0.05

There were a total of 1380 DEGs captured in both comparisons, which represents 45% of all DEGs from T3-vs-T0 and 70.4% from T12-vs-T0. Among these, 714 genes were upregulated and 666 were downregulated (Fig. 2C-D). The most differentially expressed genes (|logFC| > 3) including *SMPD3, ALOX15, GATA1*, *CCR3, GPAT2, GRO1* had higher expression in T12-vs-T0 than in T3-vs-T0, whereas *PDILIM1, FOS, LOC107133088, LOC514978, FCRL6* had higher expression in T3-vs-T0 than in T12-vs-T0. The vitamin K epoxide reductase complex subunit like 1 (*VKORC1L1*) was the most significant DEG in both comparisons (FDR < 5.4E-06). Interestingly, a relevant positional candidate gene for tick resistance *RIPK2* [24] was found downregulated (logFC _T12vsT0_ = − 0.27, FDR_T12vsT0_ = 0.0026) in T12-vs-T0. Related to this, the interacting serine/threonine kinase 1 (RIPK1; logFC = − 0.3, FDR < 0.02) and gasdermin D (GSDM; logFC = − 0.4, FDR < 0.02) genes [39] were also found to be significant and downregulated in both timepoint comparisons, and gasdermin E (GSDE, logFC _T3vsT0_ = − 1.2, FDR_T3vsT0_ = 0.0024) downregulated only in T3-vs-T0 comparison.

### Over-represented terms and biological pathways in response to tick infestation

**Fig. 3:**
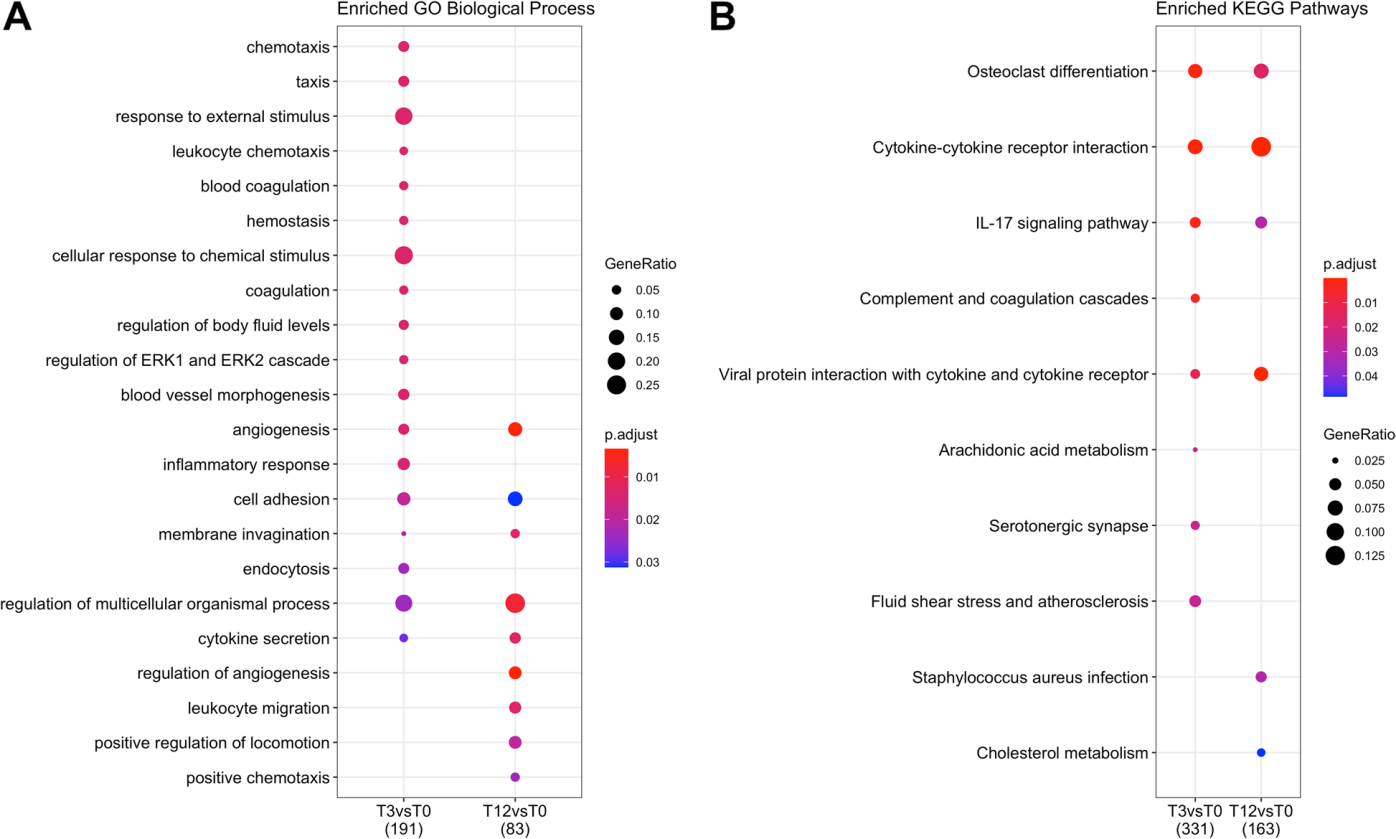
Functional enrichment analysis of the differential expression analysis between tick-exposed and tick-naïve steers. **A** Enriched GO Biological Process terms and **B** Enriched KEGG pathways in the differentially expressed genes (DEGs) from the comparison T3-vs-T0 (3-week tick-exposed vs. tick-naive) and T12-vs-T0 (12-week tick-exposed vs. tick-naive) performed by *clusterProfiler*. The dot colour represents significance (p-adjusted < 0.05) of the term, and dot size (GeneRatio) represents the ratio of input genes that are annotated in a term

Over-representation analysis of Gene Ontology (GO) terms and Kyoto Enrichment of Gene and Genomes (KEGG) pathways was performed to identify the specific biological functions associated to the DEGs detected in the comparisons between tick-exposed (3-week and 12-week) and tick-naïve steers. The GO biological process (BP) functions that were significantly enriched (p-adj < 0.05) in response to tick infestation were related to cell chemotaxis, blood coagulation, angiogenesis, inflammatory response, cell adhesion and cytokine secretion (Fig. 3A). Additionally, the KEGG enrichment analyses (p-adj < 0.05) showed that pathways including osteoclast differentiation (bta04380), cytokine-cytokine interaction (bta04060), and the IL-17 signalling pathway (bta04657) were significantly changed in host leukocytes upon tick infestation (Fig. 3B). For instance, the complement and coagulation cascade (bta04610), and arachidonic acid metabolism (bta00590) pathways were more significantly enriched in 3-week than in the 12-week exposure period. Conversely, the pathways *Staphylococcus aureus* infection (bta05150) and cholesterol metabolism (bta04979) were more enriched in the 12-week period than in the 3-week exposure period.

**Fig. 4:**
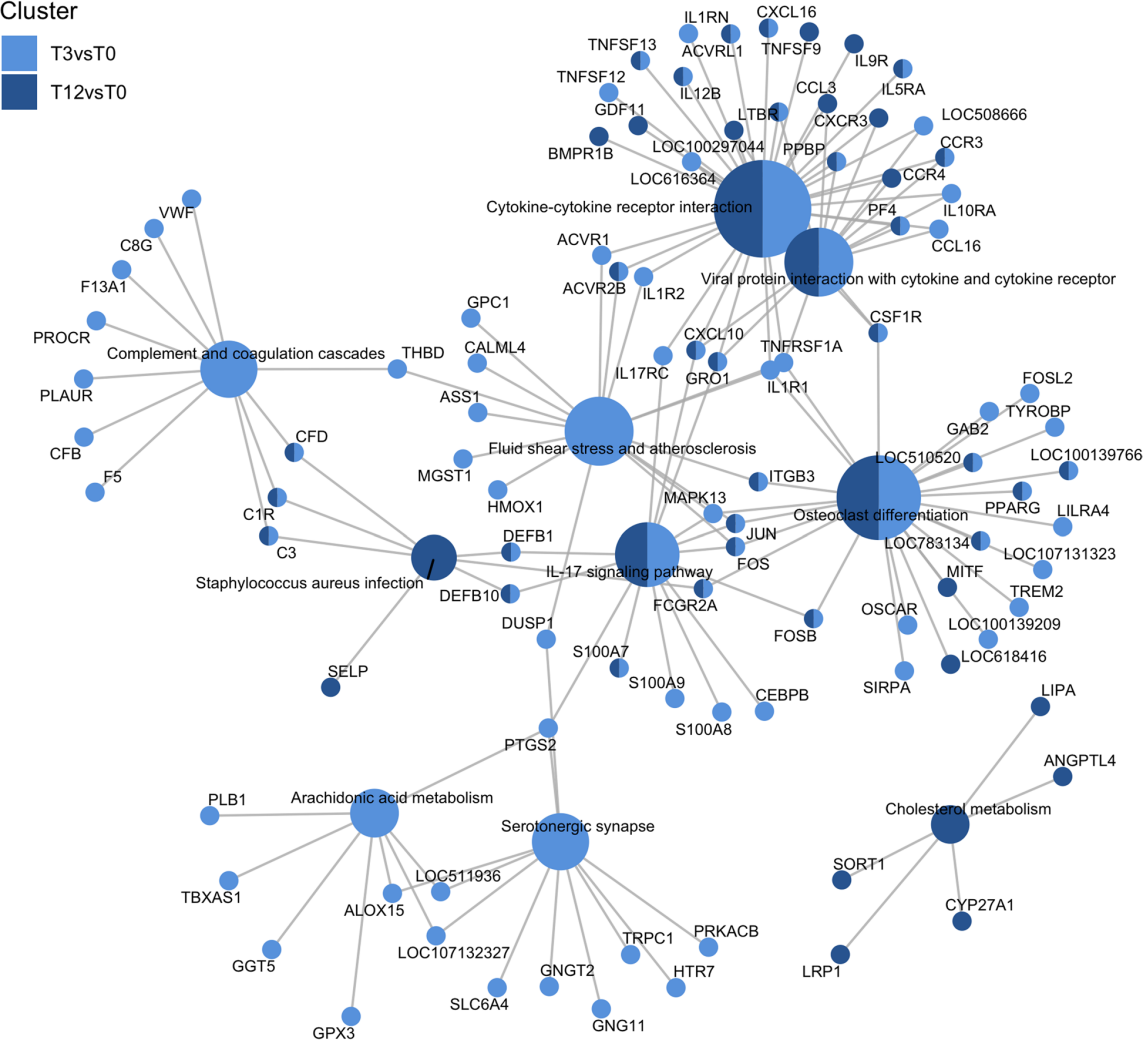
Top enriched biological pathways in tick-exposed steers. Network plot of the enriched KEGG pathways (p-adjusted < 0.05) in the differentially expressed genes (DEGs) from the comparison T3-vs-T0 (3-week tick-exposed vs. tick-naive) and T12-vs-T0 (12-week tick-exposed vs. tick-naive). Light blue = enriched in T3-vs-T0; dark blue = enriched in T12-vs-T0)

The DEGs annotated in the top 5 over-represented KEGG pathways are shown in Fig. 4. The highly downregulated genes *FOS* and *GRO1*, *FOSB* and *JUN* genes were common to various pathways including the IL-17 signalling pathway, osteoclast differentiation and cytokine-cytokine receptor interaction pathway. Highly upregulated genes such as *PROCR* and *ALOX15* were annotated in the complement and coagulation cascades and arachidonic acid metabolism pathway, respectively. A cluster of upregulated genes in the cytokine-cytokine receptor interaction pathway included *CXCL10*, *PPBP*, *PF4*, *CCR3*, *IL5RA* in short-term tick exposure, and *CCR3*, *CCR4*, *IL9R*, *BMPR1B* and *LOC100297044* in long-term tick exposure. The category network plots showing the fold changes of the featured genes in the enriched pathways are provided in Additional File 7.

**Fig. 5:**
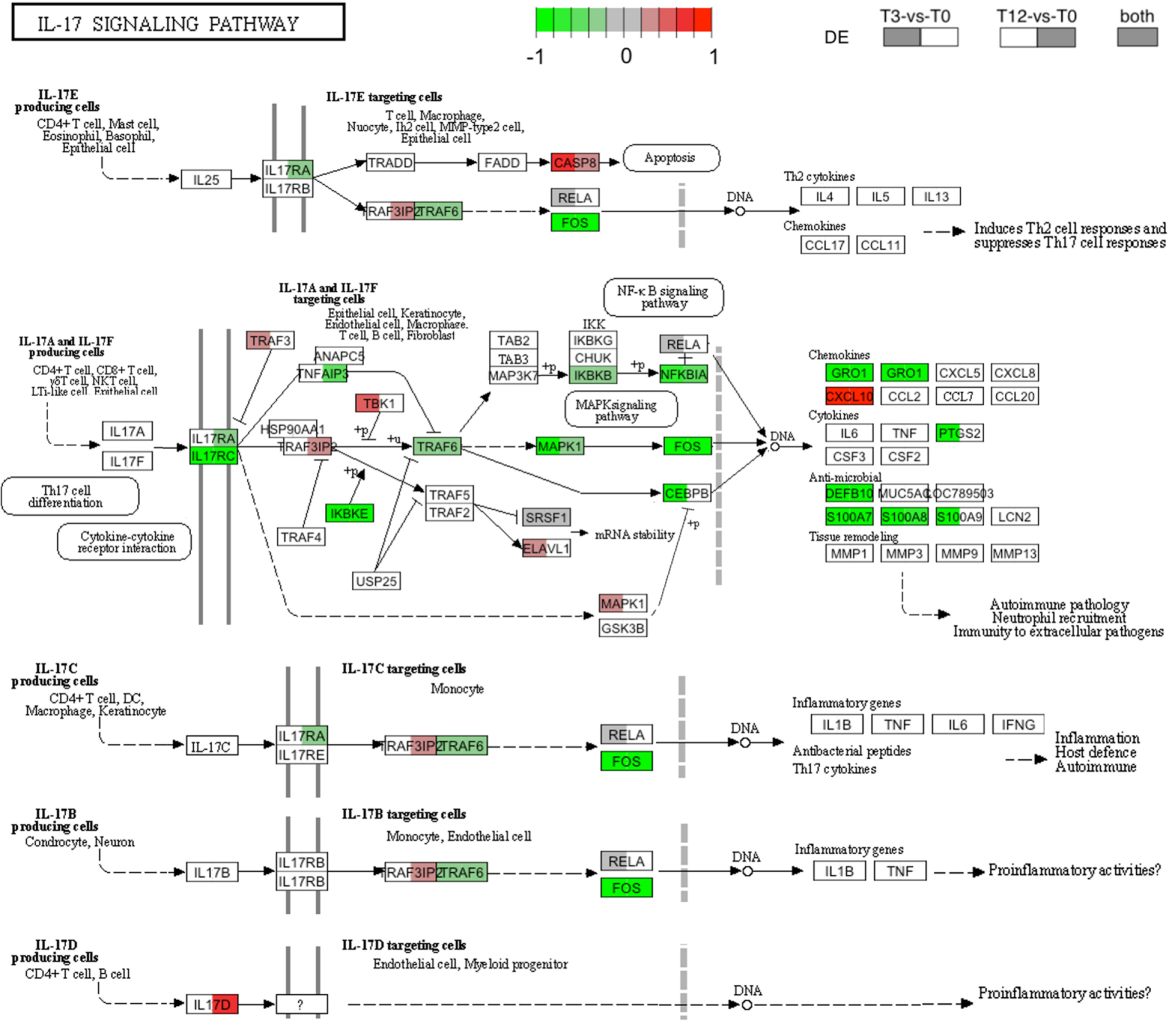
Gene expression changes in the IL-17 signalling pathway. Representation of the differentially expressed genes that were enriched in the IL-17 signalling pathway (KEGG: bta04657). Red and green boxes indicate the up- and downregulated genes in response to tick infestation, respectively. Left-side coloured box represents a gene with differential expression in the T3-vs-T0 comparison (3-week tick-exposed vs. tick-naive). Right-side coloured box represents a gene with differential expression in the T12-vs-T0 comparison (12-week tick-exposed vs. tick-naive). Fully coloured box indicates a gene that was changed in both comparisons. Blank box indicates no changes in expression. Pathway data was sourced from the KEGG database [40–42] and rendered with the *Pathview* R package

Visualization of pathway enrichment results with the *Pathview* R package demonstrated that several components of IL-17 signalling pathway were primarily downregulated by tick infestation, including *IL17RC, IL17RA, IKBKE, TRAF6, MAPK1, FOS* resulting in the repression of chemokine genes *GRO1*, cytokine genes *PTGS2*, antimicrobial genes *DEFB10, S100A7, S100A8, S100A9*, except for a few genes including *CASP8*, *TBK1* and *CXCL10* which were activated at either or both stages of the infestation (Fig. 5). Additional KEGG maps featuring DEGs for the pathways represented in Fig. 4 are provided in Additional File 8.

### Differentially expressed genes associated with divergent phenotypes of host resistance to tick infestation

Leukocyte gene expression changes between low (LR) and high (HR) host resistance steers were investigated at timepoints T0 (tick-naïve), T3 (3 weeks post-initial infestation), and T12 (12 weeks post-initial infestation). The three comparisons performed were: LR_T0_ (*n* = 3) vs. HR_T0_ (*n* = 5), LR_T3_ (*n* = 5) vs. HR_T3_ (*n* = 5), and LR_T12_ (*n* = 5) vs. HR_T12_ (*n* = 5).

**Fig. 6:**
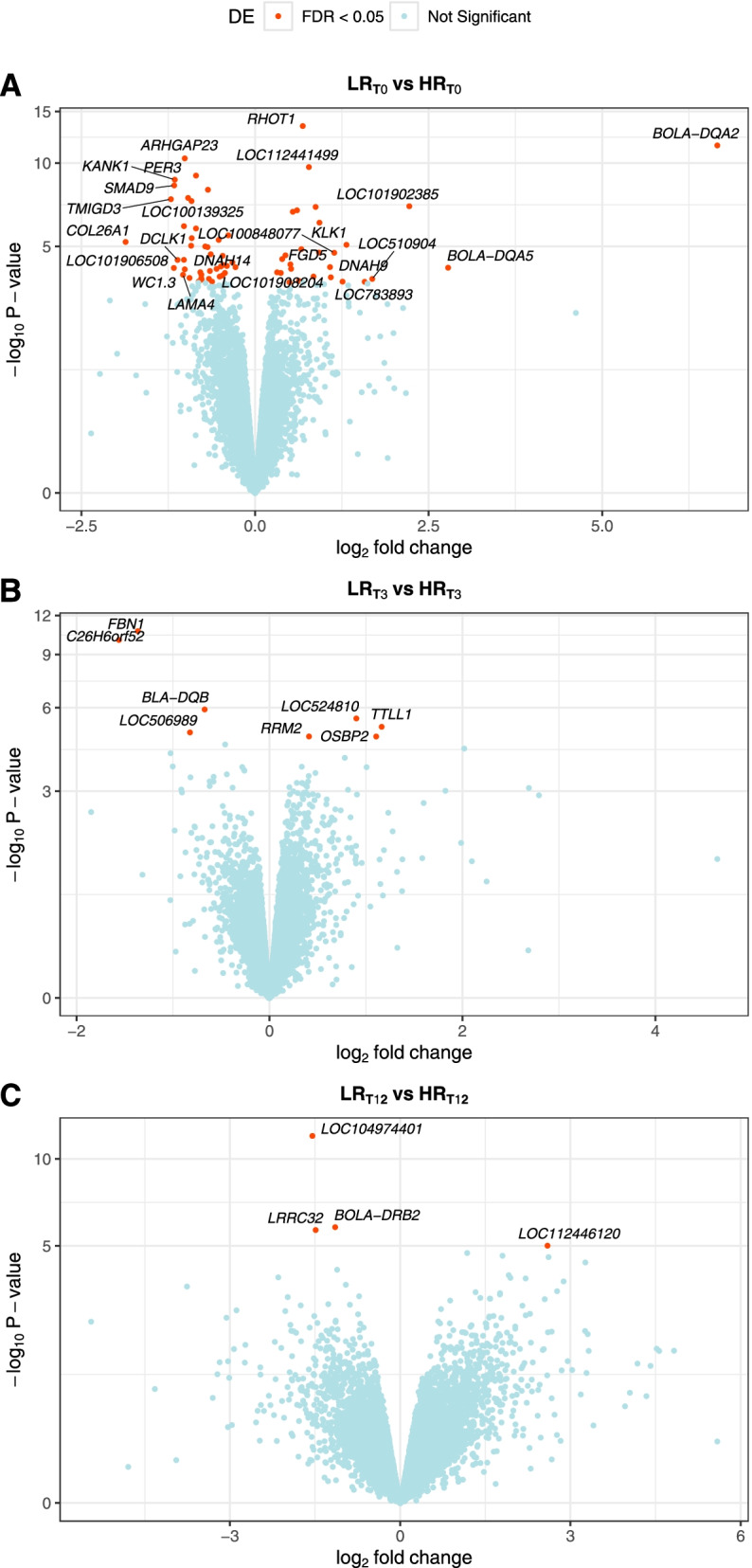
Differentially expressed genes (DEGs) in the leukocytes of low (LR) compared to high (HR) host resistance Brangus steers. **A** Volcano plot of DEGs at T0 (tick-naive) timepoint. **B** Volcano plot of DEGs at T3 (3 weeks post-initial infestation) timepoint. **C** Volcano plot of DEGs at T12 (12 weeks post-initial infestation) timepoint. The x-axis represents fold change and y-axis represents statistical significance. Symbols are shown DEGs with fold change |logFC| > 1 and FDR < 0.05 in T0, and for the DEGs in T3 and T12 (FDR < 0.05)

The total number of expressed genes tested by *edgeR* was 14,326 in T0, 13,757 genes in T3, and 14,060 genes in T12. Based on a significance threshold of FDR < 0.05, there were 69 DEGs (27 upregulated, 43 downregulated) in T0, 8 DEGs (4 upregulated, 4 downregulated) in T3, and 4 DEGs (1 upregulated, 3 downregulated) in T12 (Fig. 6; Table 3). A total of 75 unique DEGs were identified in the comparison between LR and HR steers across the three timepoints, with only 3 common genes between T0 and T3 (*LOC524810*, *FBN1* and *C26H6orf52*). Furthermore, the gene expression fold changes were higher at pre-infestation compared to the other two post-infestation timepoints. Genes of the bovine leukocyte antigen (BoLA) complex, such as *BOLA-DQA2, BOLA-DQA5* were downregulated in LR steers prior to tick exposure, whereas *BLA-DQB* and *BOLA-DQRB2* appeared upregulated in LR compared to HR steers after tick exposure at T3 and T12, respectively (Table 3; Additional File 9). A gene of interest, kallikrein-1 (*KLK1*) was found to be upregulated in LR steers (logFC =1.3; FDR =5.3E-03) before tick infestation.

**Table 3:** Significant DEGs from the comparison between low and high host resistance steers at different timepoints

Time point^a^	Symbol	Entrez ID	Name	Log_2_FC^b^	p-val	FDR^c^
T0	*BOLA-DQA2*	282,535	major histocompatibility complex, class II, DQ alpha 2	6.66	3.2E-12	2.3E-08
	*BOLA-DQA5*	282,494	major histocompatibility complex, class II, DQ alpha 5	2.78	7.3E-05	2.4E-02
	*LOC101902385*	101,902,385	uncharacterized LOC101902385	2.22	9.2E-08	1.1E-04
	*LOC510904*	510,904	uncharacterized LOC510904	1.69	1.8E-04	4.2E-02
	*DNAH9*	790,891	dynein axonemal heavy chain 9	1.58	2.3E-04	4.8E-02
	*KLK1*	493,738	kallikrein 1	1.32	8.5E-06	5.3E-03
	*LOC783893*	783,893	ankyrin repeat domain-containing protein 26-like	1.26	2.3E-04	4.8E-02
	*LOC100848077*	100,848,077	zinc finger protein 665-like	1.14	1.9E-05	9.2E-03
	*LOC101908204*	101,908,204	uncharacterized LOC101908204	1.09	1.6E-04	4.0E-02
	*FGD5*	539,766	FYVE, RhoGEF and PH domain containing 5	1.08	6.9E-05	2.4E-02
	*ARHGAP23*	523,030	Rho GTPase activating protein 23	−1.01	7.4E-07	2.0E-07
	*WC1.3*	514,534	WC1.3 molecule	−1.01	1.8E-05	2.5E-02
	*LOC100139325*	100,139,325	zinc finger protein 585A	−1.02	1.0E-07	9.4E-04
	*DNAH14*	516,576	dynein axonemal heavy chain 14	−1.02	1.5E-04	1.5E-02
	*LAMA4*	529,670	laminin subunit alpha 4	−1.04	2.1E-10	3.5E-02
	*DCLK1*	613,449	doublecortin like kinase 1	−1.12	4.1E-14	1.5E-02
	*KANK1*	534,869	KN motif and ankyrin repeat domains 1	−1.16	1.3E-05	4.3E-06
	*SMAD9*	540,806	SMAD family member 9	−1.16	2.1E-04	9.2E-06
	*LOC101906508*	101,906,508	uncharacterized LOC101906508	−1.17	1.6E-07	2.4E-02
	*TMIGD3*	104,564,307	transmembrane and immunoglobulin domain containing 3	−1.21	1.9E-07	5.0E-05
	*COL26A1*	617,340	collagen type XXVI alpha 1 chain	−1.86	8.0E-05	4.1E-03
T3	*TTLL1*	539,530	tubulin tyrosine ligase like 1	1.16	6.9E-06	1.9E-02
	*OSBP2*	510,311	oxysterol binding protein 2	1.11	1.7E-05	2.9E-02
	*LOC524810*	524,810	IgM	0.90	3.0E-06	1.0E-02
	*RRM2*	508,167	ribonucleotide reductase regulatory subunit M2	0.41	1.7E-05	2.9E-02
	*BLA-DQB*	539,241	MHC class II antigen	−0.67	1.2E-06	5.5E-03
	*LOC506989*	506,989	mitochondrial ribosomal protein L17-like	−0.82	1.2E-05	2.7E-02
	*FBN1*	281,154	fibrillin 1	−1.36	2.0E-11	2.7E-07
	*C26H6orf52*	112,444,525	chromosome 26 C6orf52 homolog	−1.56	9.3E-11	6.4E-07
T12	*LOC112446120*	112,446,120	small nucleolar RNA U2–19	2.60	9.8E-06	3.5E-02
	*BOLA-DRB2*	538,700	histocompatibility complex, class II, DR beta 2	−1.15	1.5E-06	9.4E-03
	*LRRC32*	615,467	leucine rich repeat containing 32	−1.49	2.0E-06	9.4E-03
	*LOC104974401*	104,974,401	uncharacterized LOC104974401	−1.55	1.2E-12	1.7E-08

^a^ Timepoint observed during tick infestation trial with *R. australis* larvae. T0 = tick-naïve (pre-infestation); T3 = 3 weeks post-initial infestation; T12 = 12 weeks post-initial infestation

^b^ log_2_FC represents the fold change in gene expression between low (LR) and high (HR) host resistance steers at a specified timepoint

^c^ FDR represents the false discovery rate (Benjamini-Hochberg multiple test correction of the *p*-value). Table displays genes below threshold FDR < 0.05

The functional enrichment analysis did not report any significant GO terms or KEGG pathways in the lists of LR-vs-HR DEGs.

### Overlap between genes associated with host response and host resistance to infestation

Using sample sets from high and low resistant phenotypes at three timepoints of the infestation trial, this study performed overall 5 types of gene expression comparisons. As described above, these comparisons were implemented to produce lists of DEGs between tick-exposed and naïve steers (T3-vs-T0 and T12-vs-T0) and between host resistant phenotypes (LR_T0_-vs-HR_T0_, LR_T3_-vs-HR_T3_, LR_T12_-vs-HR_T12_). Differentially expressed genes that could be relevant according to the effect of tick infestation and phenotype of interest were investigated among these lists and presented in Table 4.

**Table 4:** Differentially expressed genes by tick infestation and host resistant phenotype

Symbol	Name	T3/T0^a^	T12/T0^b^	LR_T0_/HR_T0_^c^	LR_T12_/HR_T12_^d^
*IL2RB*	interleukin 2 receptor subunit beta	0.61		0.32	
*ITGB3*	integrin subunit beta 3	1.22	1.36	− 0.40	
*CXCR5*	C-X-C motif chemokine receptor 5	0.94	0.82	−0.43	
*C18H19orf18*	chromosome 18 C19orf18 homolog	−0.85		−0.55	
*LTBP1*	latent transforming growth factor beta binding protein 1	−1.42		−0.77	
*EHD3*	EH domain containing 3		0.55	−0.33	
*GPR82*	G protein-coupled receptor 82		0.66	−0.46	
*IL9R*	interleukin 9 receptor		1.33	−0.64	
*SEMA3G*	semaphorin 3G		1.30	−0.68	
*RAB27B*	RAB27B, member RAS oncogene family		1.47	−0.91	
*BOLA-DRB2*	histocompatibility complex, class II, DR beta 2	0.87	0.67		−1.15

^a^ T3/T0 represents the fold change in gene expression between 3-week tick-exposed and tick-naïve steers

^b^ T12/T0 represents the fold change in gene expression between 12-week tick-exposed and tick-naïve steers

^c^ LR_T0_/HR_T0_ represents the fold change in gene expression between low (LR) and high (HR) host resistance steers at pre-infestation

^c^ LR_T12_/HR_T12_ represents the fold change in gene expression between low (LR) and high (HR) host resistance steers at 12 weeks post-initial infestation

*ITGB3* and *CXCR5* were leukocyte-expressed genes initially downregulated in tick-naïve steers with low host resistance, which then became upregulated in all steers following 3 and 12 weeks post-initial infestation. *BOLA-DRB2* was the only gene that was upregulated by tick infestation, but that remained downregulated in low host resistance steers after 12 weeks. Other common genes such as *C18H19orf18* and *LTBP1* showed increased downregulation after 3 weeks of tick exposure, whereas the overall trend for the rest of the genes was towards upregulation in response to tick exposure.

## Discussion

Selection for host resistance to ectoparasites is a desirable approach in the beef cattle industry as part of the overall genetic improvement strategy for animal productivity and health [4, 5]. Host resistance reduces the tick burden and therefore is a measurable phenotype, but in general, tick counts or scores are difficult to obtain, which constrains the ability to obtain large numbers of animal phenotypes for genetic improvement [9]. Although there is a heritable component for differences in host resistance, the dynamics of the host immune response also have an indispensable contribution, but these remain to be well defined [43]. The advances in high-throughput transcriptomics and bovine genomic resources offer the opportunity to put forward candidate genes for genomic selection programs.

### Host resistance in Brangus cattle

This study showed that a group of naïve Brangus steers infested repeatedly with the same dose of ticks larvae exhibited divergent phenotypes of high (HR) and low (LR) host resistance after the sixth infestation (resistance at week 5, scored on week 8) which remained steady until the end of the trial. This result is broadly consistent with the infestation study in naïve Santa-Gertrudis cattle [15] which found that significant tick burden differences between resistant and susceptible hosts appeared at week 5 of their trial, although this study used tick counts instead of scores [15]. Importantly, there is no consensus regarding the timing for host resistance onset in the literature, as it will be mostly influenced by the underlying variation in immune response among individuals. Moreover, in natural infestation conditions with fewer timepoint observations spread over a long period of time, it may be more difficult to determine when an animal has acquired resistance. As such, it is highly relevant for the cattle industry to find a more objective and unbiased measure of tick resistant phenotype.

Tick resistance is moderately heritable and the proportion of *B. indicus* genetics in a breed can influence the variation in host resistance to ticks [5, 43]. A study comparing the resistance levels of Brazilian Brangus against purebred *B. indicus* (Nellore) cattle showed that under the same natural infestation conditions Brangus animals had a higher tick burden than the Nellore [44], thus attributing the resistance of the latter to the high level of indicine genetics. The Brangus breed is a composite with typically 3/8 of indicine (37.5%) and 5/8 of taurine (62.5%) composition, but a recent study of the genomic architecture of the American Brangus showed taurine composition values deviating from the expected towards 70.4 ± 0.6% [45]. The present study found that the genomic estimates of *B. indicus* content in this Australian Brangus steer population were, in general, consistent with the theoretical expectation showing a median of 40%, but there were also some individual extremes where the lowest had 25% and the highest 49% indicine composition, the latter being one of the animals classified in the highly resistant group. However, the results also showed the inverse correlation between the indicine content and the mean tick scores was not significant, which could be due to small sample size (*n* = 29, out of 30). Hence, based on this data, it cannot be concluded with certainty that no association exists between the variables tested, thus warranting further investigation with a larger animal population. At the same time, one may question whether the demonstration of an inverse correlation between average tick burden and indicine content is sufficient to explain host resistance, as it is still of great interest to determine which indicine alleles have been selected for host resistance in Brangus [46], and also whether there are functional candidate genes associated with the immunological response of the host to tick infestation.

To the best of our knowledge, this is the first study to collect timepoint transcriptome data from peripheral blood leukocytes of Brangus cattle to explore gene expression changes related to a) the host response to repeated artificial *R. australis* challenge, and b) the host resistance differences at pre- and post-infestation. The results showed that naïve steers (both HR and LR) with exposure to a short infestation period (3 weeks) produced 1.5 times more differentially expressed genes than exposure to a long infestation period (week 12). However, among the group differences within each timepoint, it was found that the number of differentially expressed genes was the highest at pre-infestation, and then decreased significantly at early and late post-infestation. Immune responses are highly complex and dynamic requiring the interplay of innate and acquired immunity, and therefore a stronger immune response is not necessarily the most protective given that it can result in immunopathology [47, 48]. It is also context-dependent, whereby the host needs to balance off the cost/benefit of eliciting immunity and removing or maintaining pathogen challenge [49]. With this into consideration, the significance of the gene expression results is discussed next.

### Effect of tick infestation on peripheral blood leukocytes’ expression of immune and inflammatory pathways

The results showed a high number of differentially expressed genes across all animals (both HR and LR) in response to the short-term and long-term tick exposure which appeared enriched in GO terms including leukocyte chemotaxis, haemostasis, coagulation, and inflammatory response. These varied biological processes are consistent with a response to tissue damage inflicted by heavy infestation and host pathology in the attempt to eliminate the burden and have been also reported in gene expression studies in blood [31, 38] and skin [16, 18]. Moreover, tick infestation altered the expression of genes that participate in the immune system (IL-17 signalling, complement and coagulation cascades), cell signalling (cytokine-cytokine interaction), development and regeneration (osteoclast differentiation). Interestingly, these pathways appeared mostly downregulated in both short and long infestation periods. Whether these expression changes are mediated by tick salivary proteins needs further research. However, another possible explanation is that pathway downregulation in the leukocytes may have resulted from a reduction of the cell populations expressing these genes when moving out of the peripheral circulation into secondary lymphoid organs and skin [30, 31].

IL-17-mediated-immunity has emerged as an important host defence mechanism against pathogens and ectoparasites [16, 18]. In this study, several components of the downstream IL-17 signalling were downregulated by tick infestation including *IL17RC*, *IL17RA, TRAF6*, *IKBKE*, *MAPK1*, *IKBKB, IKBKE, NKFBIA*, *FOS*, the chemokine gene *GRO1*, cytokine gene *PTGS2*, and antimicrobial genes *DEFB10*, *S100A7*, *S100A8*, *S100A9*. Similar findings in mice studies have been reported for the downregulation of IL-17 receptors (IL-17R) during pathogen infection [50], and the host Th17 immunity during infestation with *Ixodes scapularis* ticks [51]. Sun et al. [52] found TRAF6 (tumor necrosis factor receptor-associated 6) to be a target of the tick salivary protein Ds Cystatin, effectively resulting in downregulation of *TRAF6* in the Toll-like receptor signalling pathway. Gene expression changes in *TRAF6* in cattle skin have also been previously reported in various cattle breeds following artificial tick challenge [19, 21].

Proinflammatory cytokines and chemokine contribute to host defence and inflammation by recruiting neutrophils and T-cells to sites of pathogen invasion [53]. The migratory capacity of activated T-cells depends on their antigenic experience and type of polarization (Th1 or Th2) which stimulates the selective upregulation of their chemokine receptors [54]. High expression of the chemokine receptor *CXCR3* and ligand *CXCL10* have been implicated with Th1 cell-mediated inflammation and chronic inflammatory conditions [55], and this study found the pair *CXCR3*/*CXCL10* to be upregulated in response to long-term infestation. *CXCL10* expression has also been reported in previous blood gene expression studies in tick-infested cattle although with discrepant patterns among breeds and resistance status [31, 38, 56], suggesting a relevant role for this chemokine gene in the adaptive immune response of cattle. Other studies have also reported expression changes in genes from the cytokine-cytokine receptor pathway in skin [16] and lymph node [26] transcriptomes, as well serum proteomes [57] of cattle exposed to ticks. Although establishing similarities in the cytokine/chemokine and receptor profiles across tick infestation studies is challenging due to differences in experimental design and timepoints of observation, it is clear that cytokine responses could contribute both to protection and immunopathology to tick feeding.

The immunological relevance of *RIPK2* gene (receptor-interacting serine-threonine kinase 2) was first proposed by Porto Neto et al. [24]. The authors suggested this to be a positional candidate gene for tick resistance, demonstrating in a knockout model that *RIPK2*-deficient mice had reduced antibody production against tick salivary gland extract. Furthermore, Wang et al. [27] showed that *RIPK2* expression was downregulated in the skin of Hereford-Shorthorn cattle of high and low tick resistance when infested with *R. microplus.* Similarly, the present study found that *RIPK2*, but also *RIPK1*, were downregulated in the leukocytes of Brangus steers following long-term tick exposure, thus this further supports the relevance of this gene, also at the transcriptional level, in host responses to tick infestation. More insights about the mechanism of host defence action involving the *RIPK1* gene in a murine model of pathogen infection have been recently reported in the study by Chen et al. [39]. Here it is understood that *RIPK1* activates gasdermin D (*GSDMD*) in macrophages and the related gasdermin E (*GSDME*) in neutrophils to promote host defence. In Brangus steers, both *GSDMD* and *GSDME* genes were also found to be downregulated by tick challenge, which could represent a reduction in the host innate immune signalling and therefore explain the overall enhanced suppression of host defence which benefits tick survival. Whether there is a role of tick salivary proteins in the downregulation of *RIPK1* and gasdermin genes should be verified by future research.

### Before tick infestation HR and LR steers reveal differences in expression of immune, tissue remodelling, and angiogenesis genes

This study found that leukocyte gene expression differences between HR and LR steers were the highest in the absence of tick challenge, which supports the hypothesis of genetic variations influencing the outcome of host resistance phenotypes among animals of the same breed. The surprising finding that gene expression profiles remain largely unchanged in the presence of tick burdens is in stark contrast to the previous findings of altered host defence responses to the cumulative effect of tick infestation. Several different factors may have contributed to the reduction of the leukocyte expression signals between phenotypes at post-infestation: rapid immune response signal (< 24 h), immunopathology interference with resistance, removal of circulating inflammatory cells by apoptosis or transmigration to the skin, immunosuppressive effects by tick feeding. The lack of temporal expression studies looking at several timepoints in the progression of bovine hosts from naïve to resistant states, as well as expression profiling of isolated immune cells, currently limits the ability to assess all of the factors mentioned previously. However, a similarity was found in a gene expression study in the lymph node of Bonsamara cattle, where there was a significant reduction in the number of DEGs in tissue infested with *R. microplus* adult ticks compared to the larval stage, concluding that lymphocyte maturation signals may be suppressed [26]. The number of animals in the group comparisons of both studies was small and therefore any implications of the commonalities found here require further investigation, where possible, in larger animal cohorts with consideration to the analysis of multiple host tissues and additional timepoints.

A key finding of this study was that observation of genes of the major histocompatibility complex (MHC), also known as bovine leukocyte antigen region (BoLA) had altered expression between HR and LR animals at pre- and post-infestation. The MHC of cattle is located in the bovine chromosome 23 and is highly polygenic and polymorphic, which allows for a wide expression repertoire of cell-surface glycoproteins that are essential for antigen processing and presentation to T cells, thus linking the innate and adaptive immune responses [58]. Several studies have reported the association of allelic diversity in BoLA genes and tick resistance/susceptibility, particularly in the class II DRB3 locus (*BOLA-DRB3*) [59–62]. In this study, the genes *BOLA-DQA2* and *BOLA-DQA5* presented as highly upregulated in LR compared to HR steers at pre-infestation, whereas after 3 weeks of repeated infestation, *BOLA-DQB* was found to be upregulated in HR animals. Additionally, *BOLA-DRB2* is one of the genes that was found to increase in expression following repeated tick exposure in both phenotypes, but after 12 weeks of tick exposure, its expression remained higher in HR than in LR steers. In this study, there was no specific association of *BOLA-DRB3* expression with resistance phenotypes, but it was observed to be upregulated in both phenotypes in response to short-term tick exposure. The contribution of these genes to tick resistance in Brangus cattle can be further explained by the findings of Goszczynski et al [46], which revealed signatures of positive selection towards indicine (Brahman) variants on the BoLA region of a Brangus population, including the location of *DQA2, DQA5, DQB, DRB2, DRB3* genes. Therefore these genes should be considered as relevant candidates in host resistance to ticks.

In regards to the most relevant DEGs detected at pre-infestation, HR steers displayed increased expression of the chemokine receptor *CXCR5* (C-X-C motif chemokine receptor 5) and ligand *CXCL12* (C-X-C motif chemokine ligand 12), tissue remodelling genes such as *FBN1* (fibrillin 1), *COL26A1* (collagen type XXVI alpha 1 chain), and cell adhesion genes including *ITGB3* (integrin subunit beta 3) and LAMA4 (lama subunit 4). *CXCR5*, which also was upregulated in response to tick infestation, is typically expressed by mature B cells and participates in the recruitment of naïve B cells into the lymph node, whereas *CXCL12* promotes leukocyte chemotaxis [63]. *FBN1* encodes an extracellular matrix glycoprotein and mutations in this gene are associated with deficient protein synthesis and weakening of connective tissue by perturbing microfibril assembly and function [64]. *COL26A1* encodes a collagen type XXVI, although it has not yet been fully characterised in cattle, mice studies suggest it differentiates from other fibrillar collagen family subgroups and functions as an extracellular matrix component [65]. ITGB3, also upregulated following long-term tick exposure and enriched in the osteoclast differentiation pathway, encodes the beta3 subunit of the integrin heterodimer which is a cell adhesion receptor that mediates the attachment of cells to the extracellular matrix and signalling in osteoclast adherence to bone [66]. Integrin can stimulate changes in intracellular calcium levels, and an increase in the expression of calcium genes has been previously reported in skin of tick-infested cattle and linked to mechanisms of host resistance [36, 67]. LAMA4 expresses alpha4 subunit of laminins which are extracellular glycoproteins with an important role in the regulation of endothelial cell adhesion and this subunit is a major structural component of the basement membrane of developing blood vessels via complex interaction with integrins [68, 69]. In this way, transcriptional changes around cytokine signalling, tissue homeostasis and angiogenesis could explain how animals may be able to better resist tick infestation and these results correlate well with previous findings in skin transcriptome studies [27].

Conversely, susceptibility to tick infestation in Brangus steers could be related to a maladaptive response due to decreased transcriptional levels of the previously discussed DEGs, while also reporting high expression of immune BoLA genes. Before tick exposure, the LR Brangus also reported increased expression of *KLK1* (kallikrein 1) and various immunoglobulin-like genes such as *LOC618463* (sialic acid-binding Ig-like lectin 13 / *SIGLEC12*), *LOC112441499* (Vlambda1) and *LOC100847119* (immunoglobulin lambda-1 light chain-like), as well as *LOC524810* (IgM). *KLK1* encodes a serine protease involved in the kallikrein-kinin system and has been implicated in proinflammatory activities but also assigned a protective role in skin wound healing [70]. While More et al. [16] reported *KLK1* to be enriched in the skin of tick resistant Braford cattle, this study found downregulated expression of *KLK1* in the leukocytes of resistant Brangus. The discrepancies in the direction of expression change could be due to differences in the tissues analysed and breed-related differences. Additionally, a higher expression of humoral immunity molecules in the skin of LR compared to HR cattle has also been reported by Wang et al. [27], however, it follows previous findings that humoral activity might not necessarily protect against tick infestation because it could contribute to immunopathology for the susceptible hosts [15].

Although much of the previous work on tick resistance was initially dedicated to the quantification of gene expression differences between *B. indicus* and *B. taurus* breeds, many of the conclusions are breed-specific, suggesting that genetic determinants of the bovine immune function still need to be properly characterised [71]. On the other hand, crossbred models are more likely to minimise breed-related effects, but as reported in this study, there is a wider variation of the individual responses to tick infestation suggesting that more experimental timepoints throughout the experimental trial should be evaluated. It can be appreciated from previous studies that exposure protocols need to be further standardised across studies and the initial exposure state of the reference herds adequately controlled for, i.e. tick-naïve vs. tick-exposed pre-treated with acaricides to determine the contribution of the innate and adaptive immune responses, as well as the many effector mechanisms linking the two. Although the sample herds were relatively small for this experimental approach, this study presents an advantageous opportunity in the recruitment of tick-naïve cattle for elucidating some of the biology behind host responses to tick infestation. While regulatory changes at the transcriptional level may be immediate and short-lived, particularly following tick attachment, the comparisons performed with the chosen timepoints have captured important information that could contribute to improving the experimental design of future artificial tick infestation trials. Lastly, future research should aim toward meta-analyses across tissues and breeds to identify and validate candidate genes for tick burden in large animal populations to promptly build reference populations for genomic-assisted selection to mitigate the impact of cattle ticks.

## Conclusions

The present transcriptomic study is the first to evaluate the effect of tick infestation on Brangus cattle with variable phenotypes of host resistance to *R. australis.* It was found that steers exposed to *R. australis* ticks for periods of 3 and 12 weeks responded to infestation by expressing leukocyte genes related to chemotaxis, cytokine secretion, and inflammatory response which are represented in immune and inflammatory pathways. IL-17 pathway and cytokine-cytokine interaction pathway appeared to be relevant in protection and immunopathology to tick challenge. Several immune, tissue remodelling, and angiogenesis genes were detected as significantly changed between high and low host resistance steers before the initial infestation, suggesting these mechanisms are relevant in the development of resistance or susceptibility to tick challenge. However, as the timing after repeated infestation progressed, a lower number of differentially expressed genes were found between the two phenotypes. The altered expression of genes from the bovine MHC complex in highly resistant animals at pre- and post- infestation stages also supports the relevance of this genomic region for disease resilience. Overall, this study offers a resource of leukocyte gene expression data that could be further evaluated in studies of ectoparasite resistance and to develop improvements to tick resistance trait selection in composite breed cattle.

## Methods

### Animals

The study was approved by the Animal Ethics Unit at The University of Queensland (certificate number QAAFI/469/18). All methods were carried out in accordance with the relevant guidelines and regulations. All methods were performed in accordance with the ARRIVE guidelines (Animal Research: Reporting of In Vivo Experiments) [57]. Thirty Brangus steers (6–8 months old and average weight of 200 kg) with no previous exposure to the *R. australis* were sourced from a tick-free region in Australia (Morven, QLD) and transported to the University of Queensland’s Pinjarra Hills Beef Research Unit (Brisbane, QLD). Animals were vaccinated using the live trivalent tick fever vaccine containing *Babesia bovis*, *Babesia bigemina* and *Anaplasma centrale* as recommended by the manufacturer (Tick Fever Centre, Queensland Department of Agriculture & Fisheries, Wacol, Queensland) at 5 weeks prior to the commencement of the trial.

### Cattle tick infestations

**Fig. 7:**
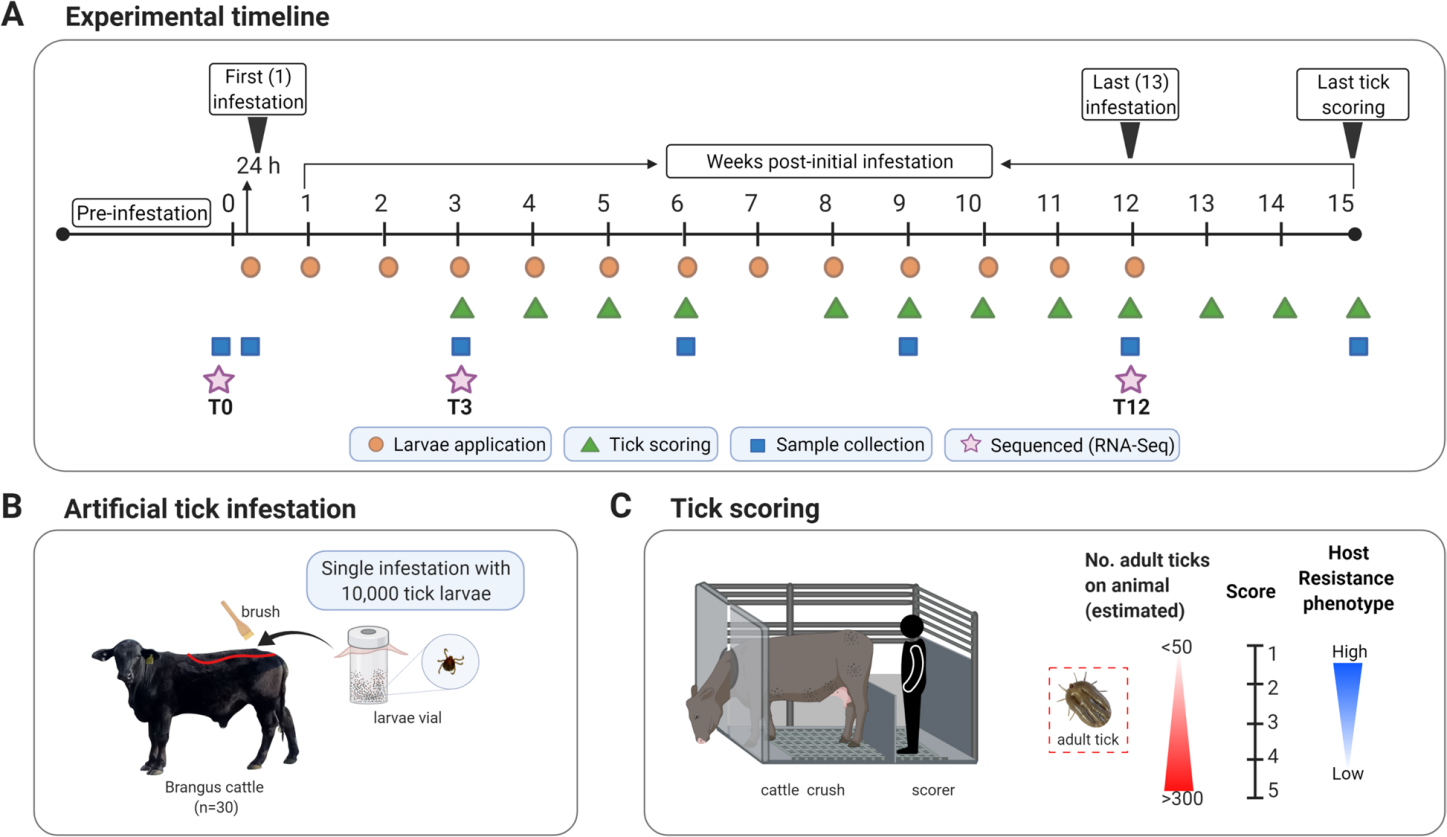
Experimental design of the study. **A** Representation of the experimental design timeline used in the artificial tick infestation trial undertaken with Brangus steers showing the frequency of larval applications (orange dots), tick scoring (green triangle), sampling (blue square), and chosen sampling timepoints for RNA sequencing (purple star). **B** Schematic of larval tick application during infestation. **C** Schematic of tick scoring method. Created with BioRender.com

The artificial infestation method was used to assess the level of host resistance to ticks on each animal [14]. Approximately 10,000 *R. australis* larvae of the non-resistant field strain [72] were applied to the base of the tail and dispersed along the back of the animal with a brush (Fig. 7A-B). Tick infestations were undertaken once weekly in the morning (7:30–8:30 am) for 12 consecutive weeks between May and August 2019 (Australian fall/winter), with a total of 13 infestations performed. The average monthly temperature during this period was 23.8 ± 0.9 °C in the Brisbane region [73].

### Tick scoring

Tick burden developing from the initial and subsequent infestations was evaluated by the same observer. Animals were scored in a cattle crush on a weekly basis period starting at week 3 until week 15 post-initial infestation (T3-T15) as represented in Fig. 7A. Scoring on week 7 could not be performed to due to staff unavailability, whereas a blank score was given in instances where animal behaviour was risky during the observation. The tick scoring scale used for this Brangus herd was as follows: 1 = 0–50 ticks, 2 = 50–100 ticks, 3 = 100–200 ticks, 4 = 200–300 ticks, 5 = more than 300 ticks, blank = not scored. Similar studies have used similar scoring methods for tick infestation phenotyping [9, 74]. The scores represent an estimation of the number of female adult ticks (un/semi/fully engorged) found on one side of the animal’s body (Fig. 7C). The mean tick score (MTS) from T8 to T15 timepoint was calculated to rank animals and define host resistance phenotype groups for transcriptomic analyses. Five animals with low MTS (1 < MTS < 2) were classified into the high host resistance (HR) group, whereas five animals with high MTS (3 < MTS < 5) were classified as low host resistance (LR) group. MTS sample values are provided in Additional File 10.

### Sample collection and leukocyte RNA extraction

Sample collection was performed before tick challenge (day 0/T0), at 6 hours post-initial infestation, and then at day 22 (T3), 44 (T6), 65 (T9) and 85 (T12) of the trial. For each animal, approximately 2–6 mL of blood were drawn from the jugular vein into a single BD Vacutainer® K_2_EDTA tube (BD, USA) and kept on ice until transported to the laboratory. Red blood cell lysis for leukocyte isolation was performed within 12–48 hours post-collection with a protocol adaptation from the miRNeasy mini kit guide “Appendix D” (QIAGEN, USA) using 1X RBC lysis buffer (168 mM NH_4_Cl; 10 mM KHCO_3_, 0.1 mM EDTA in 500 mL Milli-Q water, pH 7.4). The leukocyte pellets were homogenized with 1200 μL of QIAzol reagent (QIAGEN, USA) and stored at − 80 °C until further use.

Leukocyte RNA was isolated from animals classified as HR (*n* = 5) and LR (*n* = 5) from days 0, 21, and 85 post-initial infestation. Samples from these timepoints are referred to in this manuscript as HR/LR-T0, HR/LR-T3, and HR/LR-T12 datasets. In total, 30 samples were extracted with the miRNeasy mini kit (QIAGEN, USA) as per manufacturer’s instructions. RNA samples were treated with DNA-free™ DNAse (Life Technologies, USA) and RNA was quantified with the Nanodrop 2000 spectrophotometer (ThermoFisher, USA). RNA quality analysis (RIN) was performed with the 2100 Bioanalyzer Instrument (Agilent Technologies, USA) by the Institute for Molecular Biosciences Sequencing Facility in St. Lucia, Australia. RIN sample values are provided in Additional File 10.

### RNA sequencing

Out of the 30 RNA samples prepared for RNA-sequencing, two samples were removed due to low RNA concentration. Hence, a total of 28 cDNA libraries were prepared with the TruSeq Stranded mRNA kit (Illumina, USA) and sequenced as 100 bp single-end reads in one flow cell lane on a NovaSeq 6000 sequencer (Illumina, USA). The Illumina bcl2fastq 2.20.0.422 pipeline was used to generate the sequence data. Library preparation and sequencing were performed by the Australian Genome Research Facility (AGRF) in Melbourne, Australia.

### Genomic estimate of *Bos indicus* content (BIC)

Tail hair DNA was used to genotype all steers with the GeneSeek® Genomic Profiler™ Bovine 50 K (GGP 50 K) by Neogen Australasia in Gatton, Australia. *Bos indicus* content was calculated using the 35 K array by comparing each animal to a large purebred *B. indicus* dataset [75]. Using a GBLUP model, phenotypes were assigned as 1 for *B. indicus* and 0 if not and the effect of each SNP was back-solved [76]. Prediction equations for *B. indicus* purebred were then used to estimate BIC in the steers [75]. The Pearson correlation method was used to test the significance between MTS and BIC values obtained for 29 out of 30 Brangus samples. BIC values for DEG covariate models are supplied in Additional File 10.

### Bioinformatics pipeline for differential gene expression analysis

The bioinformatics analyses were carried out in The University of Queensland High Performance Computing Cluster [77], Galaxy Australia Server [78], and RStudio [79]. Briefly, a read quality control was performed with FastQC software (version 0.11.4) [80]. Adapters and low quality reads were removed with Trimmomatic software (version 0.35) [81] using parameters for single-end reads including –phred 33 LEADING:3 TRAILING:3 SLIDINGWINDOW:4:15 MINLEN:36 and Illumina adapter sequence “AGATCGGAAGAGC”. Reads were aligned to the genome assembly *Bos taurus* ARS-UCD1.2 using HISAT2 (Galaxy version 2.1.0 + galaxy6) [82] with default parameters and read strandness set to “reverse”. Gene count data were generated with featureCounts (Galaxy Version 1.6.4) [83] using default parameters and strand information set to “reverse”. The reference genome and annotation file for bosTau9 were obtained from the UCSC genome browser database [84].

Gene count matrices consisting of 28,787 *B. taurus* genes as rows and samples as columns were input in RStudio [79]. The *edgeR* Bioconductor package [85] was used to perform all differential gene expression analyses. Gene filtering was performed with an expression threshold of CPM > 0.5 according to n samples, where n was determined by the condition with the lowest number of samples tested. Libraries were normalised with the function *calcNormFactors* which uses the trimmed mean of M-values (TMM) method [86]. Under the negative binomial model, the common and tagwise dispersion were calculated with the function *estimateDisp*. Gene expression models for the analysis of host response to tick infestation (Model 1) and host resistance phenotypes (Model 2:A-B) are listed in Table 5 and further described next.

**Table 5:** Gene expression models and explanatory variables for leukocyte data

Model	Interpretation
Model 1: ~IW + MTS + BIC + RIN	Gene expression of tick-exposed and non-exposed groups (defined by infestation timepoint).
Model 2A: ~ MTS + BIC + RIN	Gene expression of low and high host resistance groups (defined by MTS) at pre-infestation timepoint.
Model 2B: ~MTS + TPS + BIC + RIN	Gene expression of low and high host resistance groups (defined by MTS) at post-infestation timepoints.

*Abbreviations*: *IW* infestation week timepoint, *MTS* mean tick score, *TPS* timepoint tick score, *BIC Bos Indicus* content, *RIN* sample RIN value

Experimental design data used with these models are provided in Additional file 10.

#### Model 1: host response to tick infestation

Differentially expressed genes were detected between tick-exposed (T3 or T12, *n* = 10 each) and non-exposed (tick-naive) steers (T0, *n* = 8) with the quasi-likelihood F-test (edgeRQLF) and the linear model ~IW + MTS + BIC + RIN. Here, infestation week timepoint (IW) was fitted as a factor with two levels for comparisons of timepoint datasets as T3-vs-T0 and T12-vs-T0. The covariates of mean tick score (MTS), *B. indicus* content (BIC), sample RIN value (RIN) were implemented to account for additional sources of variation in the datasets. Significant differentially expressed genes (DEGs) were identified with a false discovery rate (FDR) threshold at FDR < 5%.

#### Model 2: host resistance phenotypes

Differentially expressed genes were detected between high (HR) and low (LR) host resistance phenotypes with the likelihood ratio test (edgeRLRT) using two types of linear models: Model 2A (~MTS + BIC + RIN) implemented for pre-infestation (T0) data, and Model 2B (~MTS + TPS + BIC + RIN) implemented for post-infestation data (T3 and T12).

Model 2A evaluated the comparison of the groups LR-T0 (*n* = 5) and HR-T0 (*n* = 3) according to mean tick score (MTS) fitted as a continuous variable while accounting for the effects of *B. indicus* content (BIC) and sample RIN value (RIN).

Model 2B was the same as model 2A but also included a covariate for timepoint tick score (TPS) to account for the effect of timepoint tick burden measured on the T3 and T12 datasets, respectively. The comparisons were performed between LR-T3 (*n* = 5) and HR-T3 (*n* = 5), and between LR-T12 (*n* = 5) and HR-T12 (*n* = 5) according to the MTS value. Significant DEGs were identified with a threshold at FDR < 5%.

### Functional enrichment analysis

Over-representation analysis (ORA) of Gene Ontology (GO) terms and KEGG (Kyoto Encyclopedia Genes and Genomes Pathways) were performed with the *clusterProfiler* R package [87] using filtered gene lists (FDR < 0.05 and |logFC| > 1) from T3-vs-T0 and T12-vs-T0 datasets. Additional filtering of highly redundant parent-child terms in the ORA-GO output was applied with the *simplify* function with the default threshold value. Graphics were created with dot plot and category network plot functions from this package. Pathway graphs were rendered with the *Pathview* R package [88] for *Bos taurus* organism sourced from the KEGG database [40–42].

## Supplementary Information

**Additional file 1.**

**Additional file 2.**

**Additional file 3.**

**Additional file 4.**

**Additional file 5.**

**Additional file 6.**

**Additional file 7.**

**Additional file 8.**

**Additional file 9.**

**Additional file 10.**

## Data Availability

All relevant data are included in the manuscript and its Supplementary files. The datasets have been deposited with links to BioProject accession number PRJNA802321 in the NCBI BioProject database (https://www.ncbi.nlm.nih.gov/bioproject/PRJNA802321).

## Abbreviations

T0: Pre-infestation timepoint
T3: Week 3 post-initial infestation timepoint
T12: Week 12 post-initial infestation timepoint
HR: High host Resistance phenotype
LR: Low host Resistance phenotype
DEG: Differentially Expressed Gene
GO: Gene Ontology
KEGG: Kyoto Encyclopedia Genes and Genomes Pathways
FDR: False Discovery Rate
FC: Fold change
MTS: Mean Tick Score
IW: Infestation Week
TPS: Timepoint Tick Score
RIN: RNA Integrity Number
BIC: *Bos indicus* content
CPM: Counts per million
MHC: Major Histocompatibility Complex
