# Supplementary material for: Transcriptional changes in the peripheral blood leukocytes from Brangus cattle before and after tick challenge with Rhipicephalus australis
Source: BMC Genomics. 2022 Jun 20;23:454. doi: 10.1186/s12864-022-08686-3 (PMC9208207; doi:10.1186/s12864-022-08686-3)
Supplement: Supplementary file 1 — Additional file 1. [file 12864_2022_8686_MOESM1_ESM.pdf]

Additional File 1. Tick scoring results. Table of tick scores collected for 30 Brangus cattle artificially infested with the *Rhipicephalus australis* (cattle tick) for 13 consecutive weeks. Timepoints (T) represent the number of weeks after the first artificial tick infestation. Tick scores represent the tick burden on one-side of the body, defined as follows: 1 was 0-50 ticks, 2 was 50-100 ticks, 3 was 100-200 ticks, 4 was 200-300, and 5 was > 300 ticks and NA= non-recorded/blank score. Heatmap colours range from green to red correspond to tick scores from 1 to 5. *Denotes animal and timepoints selected for RNA-Seq analysis in this study including pre-infestation. The mean tick score (MTS) and standard deviation (SD) were calculated between timepoints T8 to T15.

Animal ID	*T3	T4	T5	T6	T8	T9	T10	T11	*T12	T13	T14	T15	Mean tick score (MTS) T8-T15	Standard deviation (SD)
*B05	2	2	1	3	1	NA	1	1	1	1	1	1	1.00	0.00
*B12	3	3	3	1	1	1	1	1	1	1	1	1	1.00	0.00
*B11	2	2	3	2	1	1	1	1	1	1	3	1	1.25	0.71
B10	3	3	3	3	NA	1	1	1	1	2	3	1	1.43	0.79
B23	2	3	3	3	1	2	1	2	1	1	2	2	1.50	0.53
B24	2	3	3	3	2	1	2	1	2	1	2	1	1.50	0.53
B03	2	3	3	4	1	2	1	1	2	1	3	1	1.50	0.76
B06	2	4	3	5	1	1	1	1	1	1	3	3	1.50	0.93
*B22	2	3	3	3	2	2	1	1	1	2	1	3	1.63	0.74
B14	3	4	3	3	1	1	3	1	2	2	2	1	1.63	0.74
*B29	1	4	1	1	1	2	4	2	1	1	2	2	1.88	0.99
B19	5	5	2	2	3	1	3	3	1	1	2	2	2.00	0.93
B02	1	3	3	NA	3	3	1	2	1	1	3	3	2.13	0.99
B01	3	3	3	2	1	3	2	3	3	2	3	3	2.50	0.76
B15	2	2	1	3	3	2	4	1	3	3	3	2	2.63	0.92
B09	2	3	1	NA	3	2	2	3	3	2	3	3	2.63	0.52
B13	2	4	3	5	3	3	1	NA	2	4	5	1	2.71	1.50
B16	1	1	NA	1	1	3	2	3	3	3	5	2	2.75	1.16
B28	3	3	4	3	2	2	2	3	1	3	5	5	2.88	1.46
B18	1	1	2	1	1	NA	4	2	3	3	3	5	3.00	1.29

**B21**	3	4	4	3	1	2	4	3	2	2	5	5	3.00	1.51
**B27**	2	3	3	4	3	3	1	3	4	3	4	4	3.13	0.99
**B32**	3	3	3	5	3	3	3	4	3	3	3	4	3.25	0.46
**B31**	3	3	1	2	4	4	2	4	4	2	5	2	3.38	1.19
***B04**	1	1	3	NA	1	4	3	4	3	4	5	5	3.63	1.30
**B08**	5	3	3	3	3	4	3	2	NA	4	5	5	3.71	1.11
***B07**	1	3	2	2	3	4	5	5	5	3	4	3	4.00	0.93
***B20**	4	5	3	4	5	5	4	5	4	3	3	4	4.13	0.83
***B17**	3	3	3	5	4	4	5		5	3	5	3	4.14	0.90
***B25**	5	5	5	3	5	5	1	5	5	5	5	5	4.50	1.41
