# Supplementary material for: Transcriptional changes in the peripheral blood leukocytes from Brangus cattle before and after tick challenge with Rhipicephalus australis
Source: BMC Genomics. 2022 Jun 20;23:454. doi: 10.1186/s12864-022-08686-3 (PMC9208207; doi:10.1186/s12864-022-08686-3)

Additional File 2: Genomic estimates of *Bos indicus* content and relationship with mean tick score in Brangus steers. A) Dot plot showing the distribution of the *B. indicus* content (%) of 29 steers with coloured dots for the animals classified into the high (red, n=5) and low (blue, n=5) host resistant phenotypes by tick scoring analysis. B) Scatter plot showing that correlation between mean tick score and *B. indicus* content (%) in the dataset of 29 steers was not significant (r= -0.17, *p*=0.37).

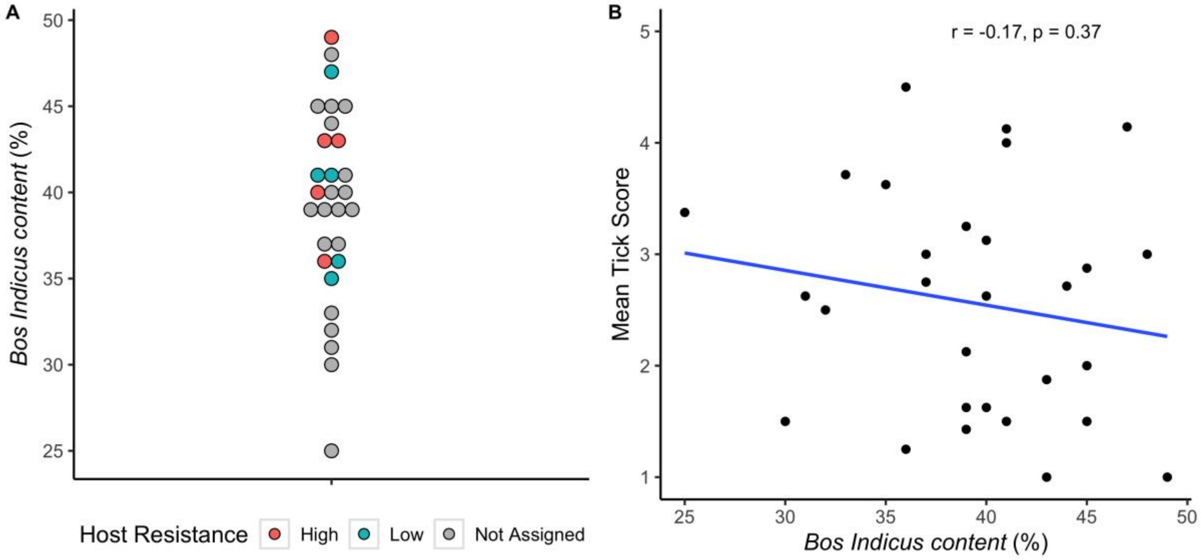

Supplement: Supplementary file 2 — Additional file 2. [file 12864_2022_8686_MOESM2_ESM.pdf]
