# Supplementary material for: Transcriptional changes in the peripheral blood leukocytes from Brangus cattle before and after tick challenge with Rhipicephalus australis
Source: BMC Genomics. 2022 Jun 20;23:454. doi: 10.1186/s12864-022-08686-3 (PMC9208207; doi:10.1186/s12864-022-08686-3)

Additional File 4: Multidimensional scaling plots (MDS) of RNA-seq samples.
MDS plots of RNA-seq samples separated in the first and second dimension. Plots are derived from the pairwise comparison between timepoints (T3-vs-T0 and T12-vs-T0) and between host resistance phenotype (LR-vs-HR) at every single timepoint. Abbreviation: T0 = pre-infestation, T3= 3-weeks post-initial infestation, T12= 12-weeks post-initial infestation, HR= high host resistance, LR =low host resistance.

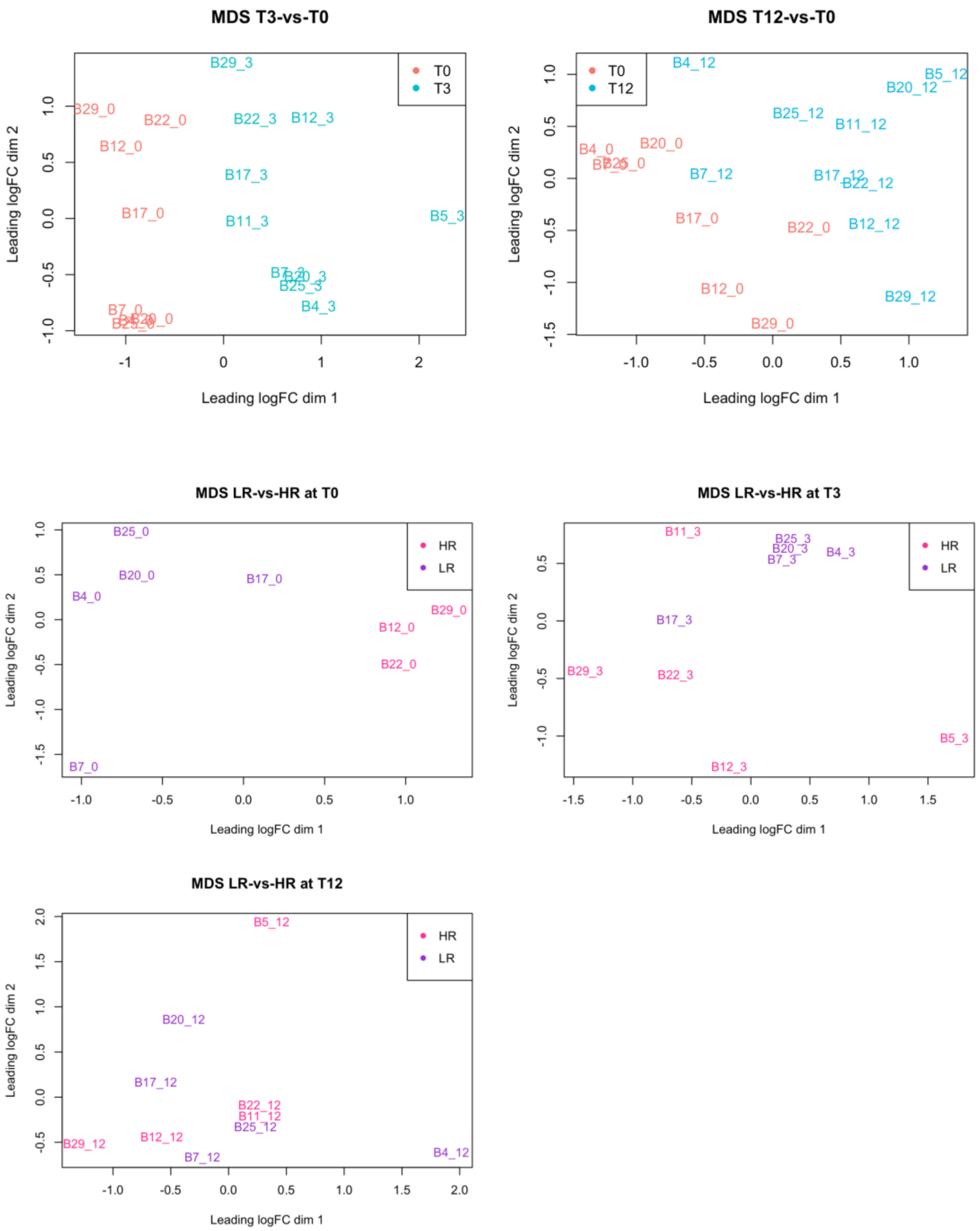

Supplement: Supplementary file 4 — Additional file 4. [file 12864_2022_8686_MOESM4_ESM.pdf]
