# Supplementary material for: Transcriptional changes in the peripheral blood leukocytes from Brangus cattle before and after tick challenge with Rhipicephalus australis
Source: BMC Genomics. 2022 Jun 20;23:454. doi: 10.1186/s12864-022-08686-3 (PMC9208207; doi:10.1186/s12864-022-08686-3)

Additional File 7: Category gene network plot of top enriched KEGG pathways with their associated up- and down-regulated DEGs. A) Plot for enriched pathways in the comparison of 3-week tick-infested vs. tick-naïve steers, B) Plot for enriched pathways in the comparison of 12-week tick-infested vs. tick-naïve steers. Gene dot represents expression fold change (red=upregulated; blue= downregulated) and category dot size represents the number of genes annotated in that term.

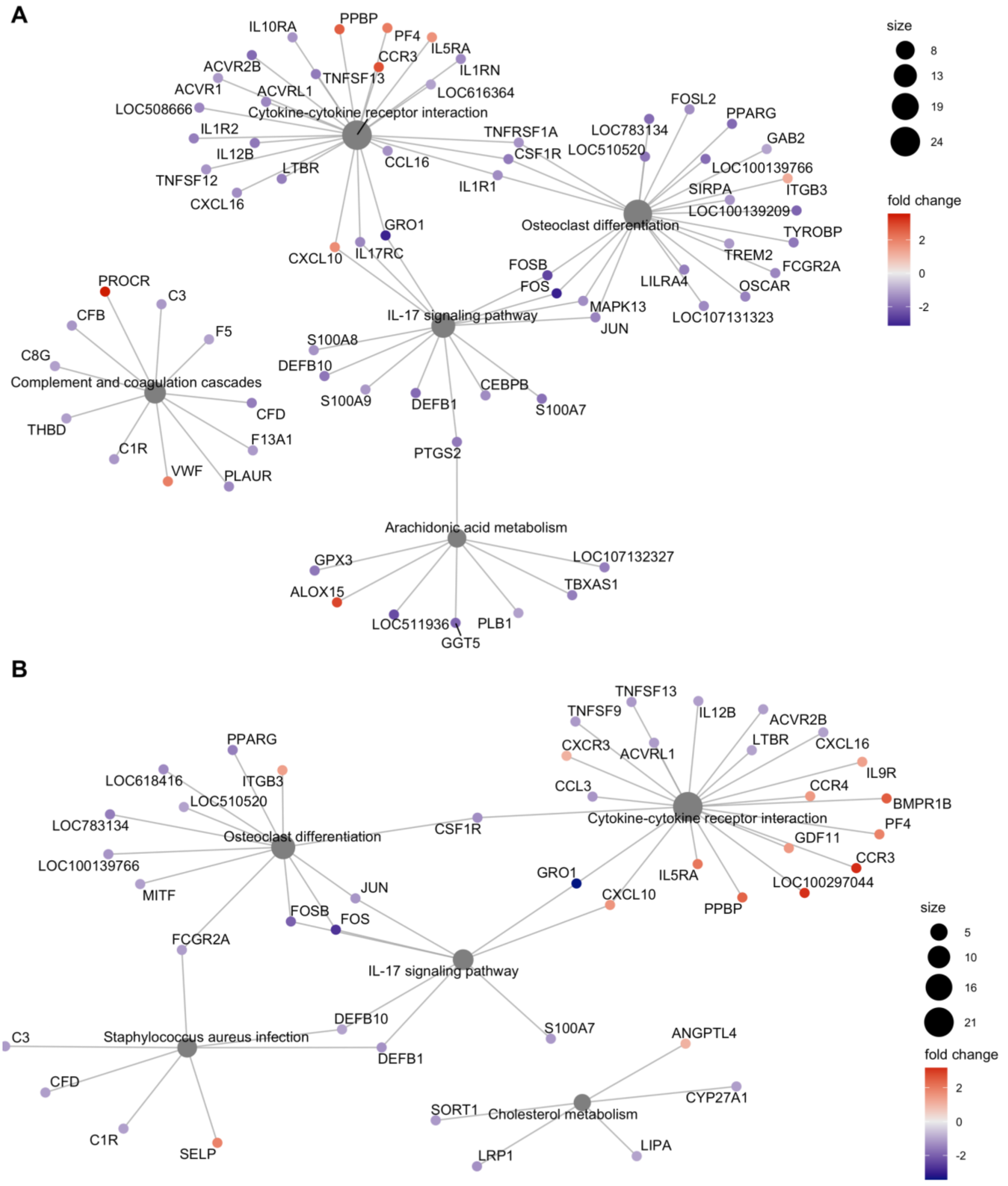

Supplement: Supplementary file 7 — Additional file 7. [file 12864_2022_8686_MOESM7_ESM.pdf]
