# Supplementary material for: Transcriptional changes in the peripheral blood leukocytes from Brangus cattle before and after tick challenge with Rhipicephalus australis
Source: BMC Genomics. 2022 Jun 20;23:454. doi: 10.1186/s12864-022-08686-3 (PMC9208207; doi:10.1186/s12864-022-08686-3)
Supplement: Supplementary file 8 — Additional file 8. [file 12864_2022_8686_MOESM8_ESM.pdf]

Additional File 8: **Significant differentially expressed genes in the leukocyte host response to tick infestation mapped to the top enriched KEGG pathways**.
Differentially expressed genes (DEGs, FDR <0.05) were obtained from the comparison of 3-week tick-infested vs. tick-naïve steers (T3-vs-T0) and 12-week tick-infested vs. tick-naïve steers (T12-vs-T0). Pathways graphs show highlighted in red the upregulated DEGs and in green the downregulated DEGs. Partially coloured box represents a DEG featuring in one of the two timepoint comparisons (left = T3-vs-T0, right= T12-vs-T0) or in both timepoint comparisons (fully coloured). Pathway data was sourced from the KEGG database [86-88] and rendered with Pathview R package for *Bos taurus* (cow) organism and depicted below as follows: IL-17 signalling pathway (bta04657), Cytokine-cytokine receptor interaction (bta04060), Osteoclast differentiation (bta04380), Complement and coagulation cascades (bta04610), Arachidonic acid metabolism (bta00590), and Cholesterol metabolism (bta04979).

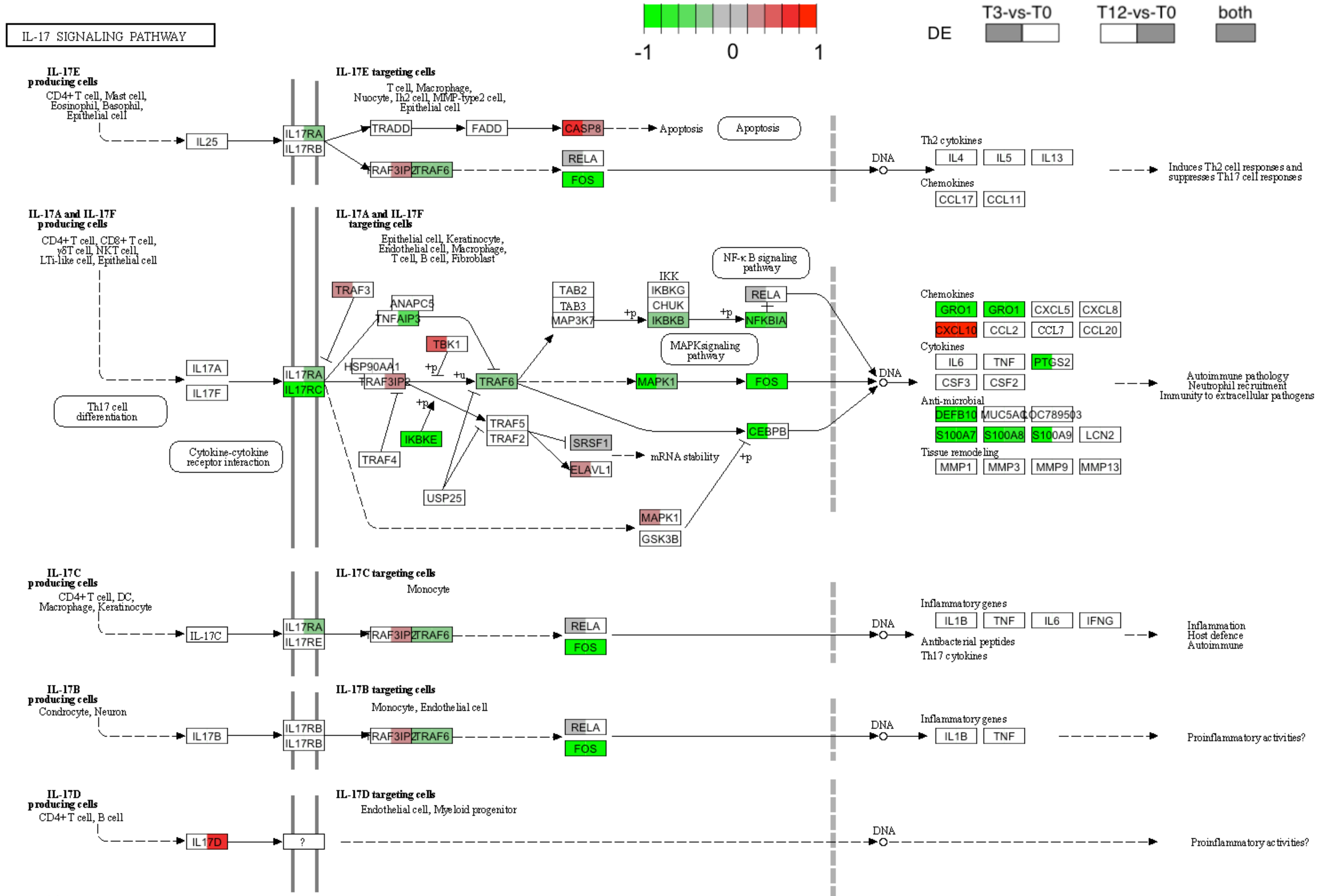

Data on KEGG graph
Rendered by Pathview

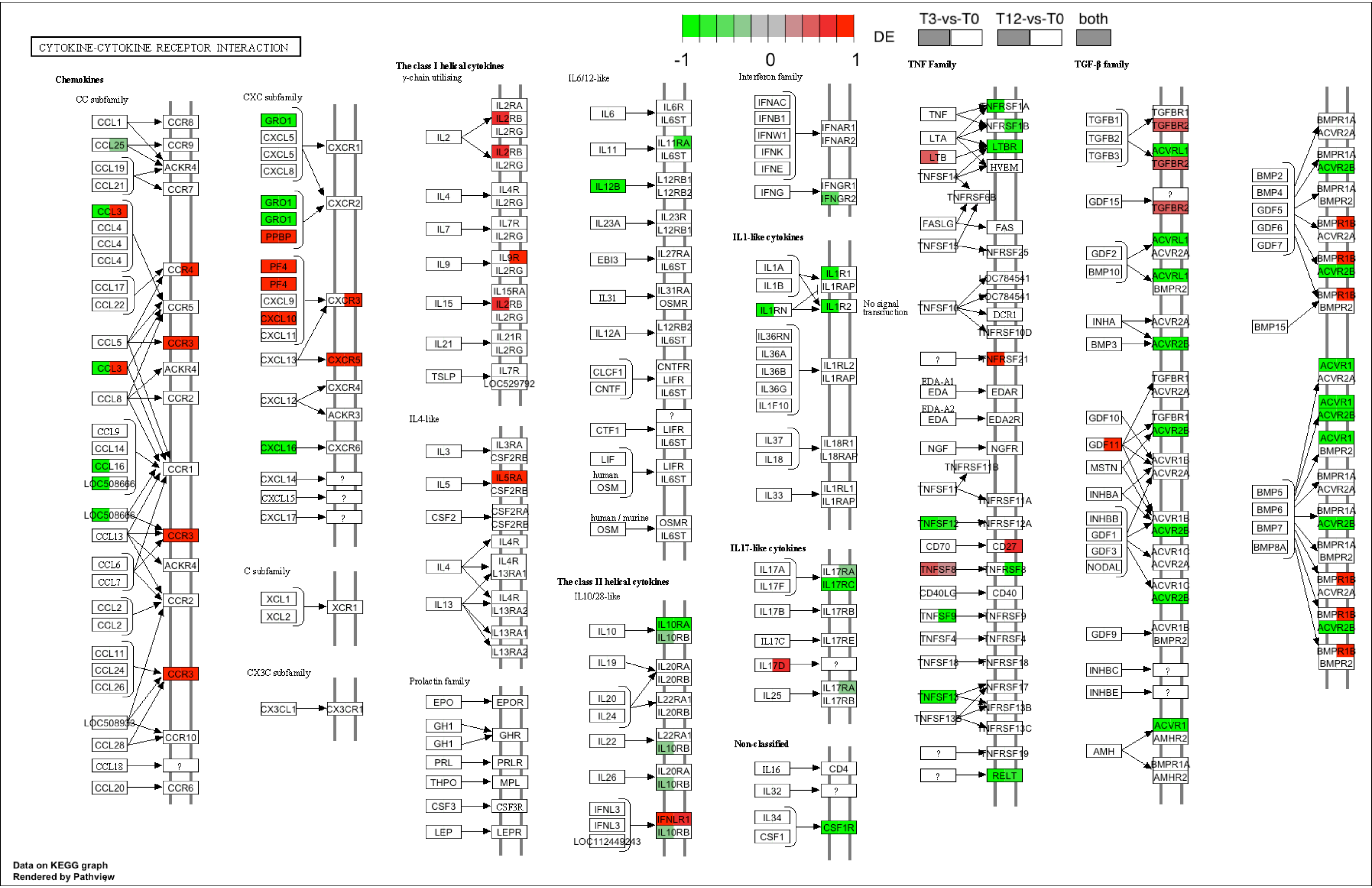
CYTOKINE-CYTOKINE RECEPTOR INTERACTION
-1
0
1
DE
T3-vs-T0
T12-vs-T0
both
Chemokines
CC subfamily
CXC subfamily
C subfamily
CX3C subfamily
The class I helical cytokines
γ-chain utilising
IL4-like
Prolactin family
IL6/12-like
The class II helical cytokines
IL10/28-like
Interferon family
IL1-like cytokines
No signal transduction
IL17-like cytokines
Non-classified
TNF Family
TGF-β family
Data on KEGG graph
Rendered by Pathview

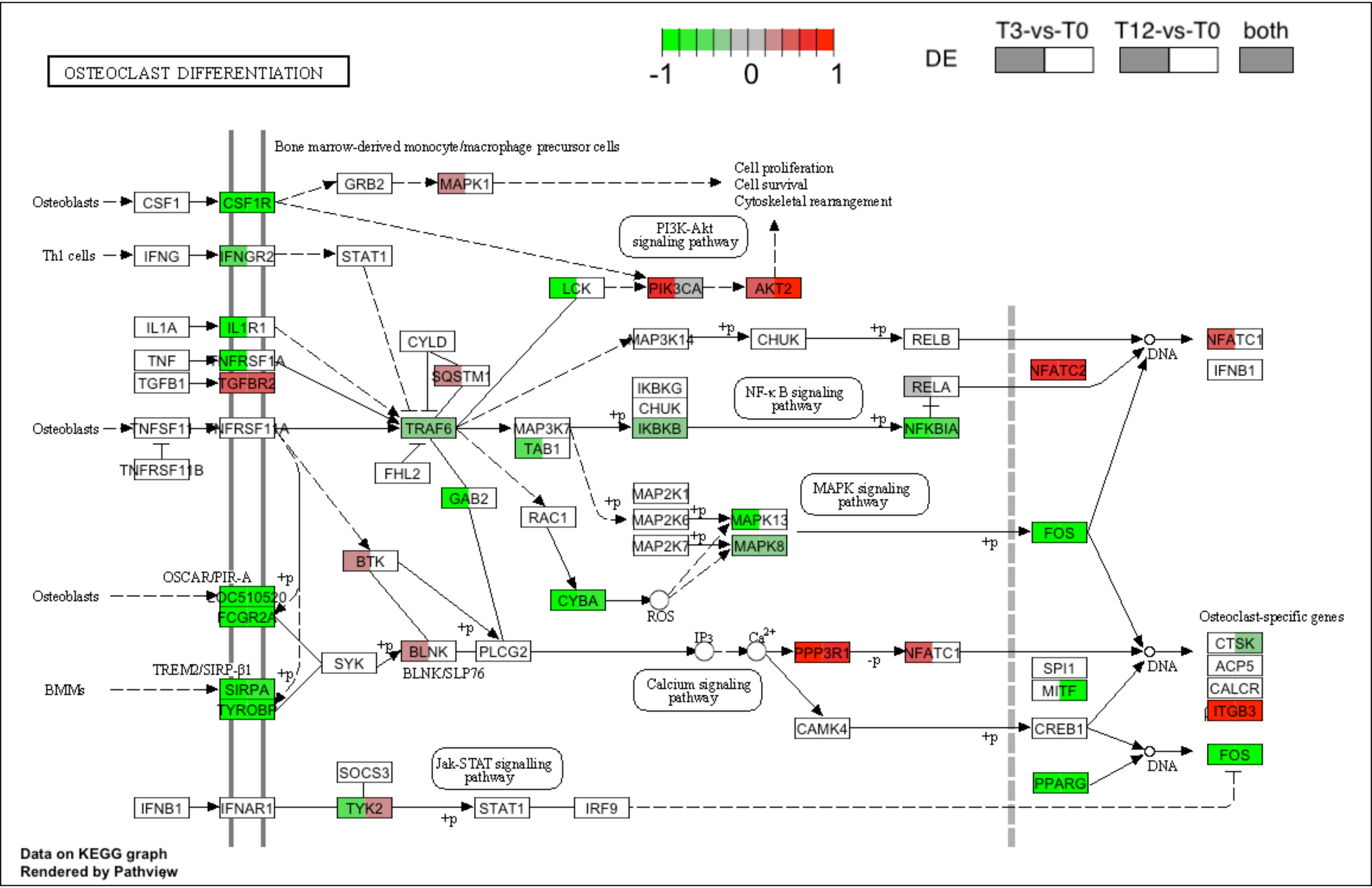
OSTEOCLAST DIFFERENTIATION
-1
0
1
DE
T3-vs-T0
T12-vs-T0
both
Bone marrow-derived monocyte/macrophage precursor cells
Osteoblasts
CSF1
CSF1R
GRB2
MAPK1
Cell proliferation
Cell survival
Cytoskeletal rearrangement
Th1 cells
IFNG
IFNGR2
STAT1
PI3K-Akt signaling pathway
LCK
PIK3CA
AKT2
IL1A
IL1R1
CYLD
MAP3K14
CHUK
RELB
NFATC1
TNF
TNFRSF1A
SQSTM1
DNA
NFATC2
IFNB1
TGFB1
TGFBR2
IKBKG
CHUK
NF-κB signaling pathway
RELA
Osteoblasts
TNFSF11
TNFRSF11A
TRAF6
MAP3K7
IKBKB
NFKBIA
TAB1
TNFRSF11B
FHL2
MAP2K1
MAPK signaling pathway
GAB2
RAC1
MAP2K6
MAPK13
FOS
MAP2K7
MAPK8
BTK
OSCAR/PIR-A
Osteoblasts
LOC510520
FCGR2A
CYBA
ROS
Osteoclast-specific genes
CTSK
ACP5
CALCR
ITGB3
BLNK
PLCG2
IP3
Ca^{2+}
PPP3R1
NFATC1
DNA
SYK
BLNK/SLP76
SPI1
MITF
TREM2/SIRP-β1
BMMs
SIRPA
TYROBP
Calcium signaling pathway
CAMK4
CREB1
DNA
FOS
SOCS3
Jak-STAT signalling pathway
PPARG
IFNB1
IFNAR1
TYK2
STAT1
IRF9
+p
-p
Data on KEGG graph
Rendered by Pathview

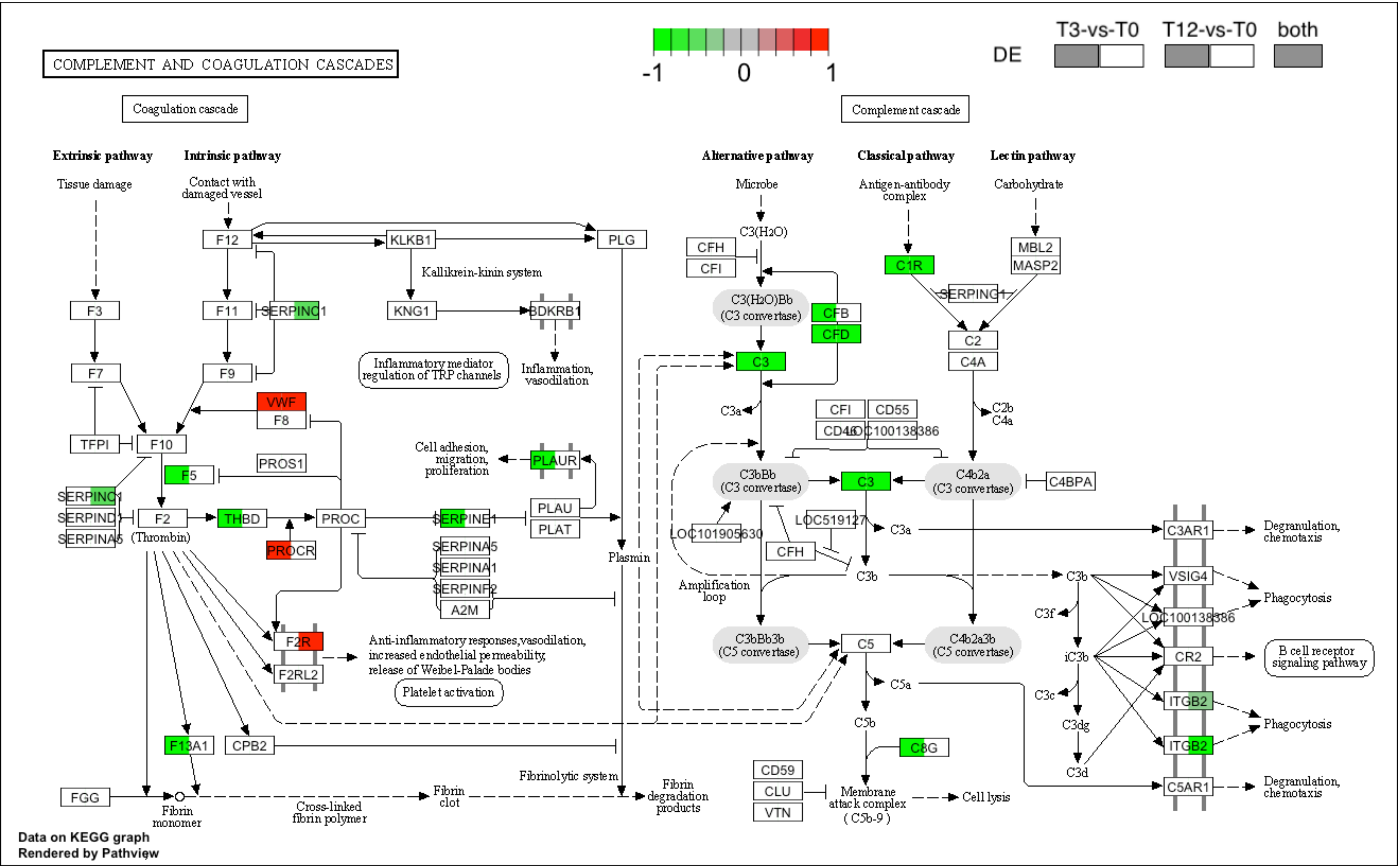

COMPLEMENT AND COAGULATION CASCADES
-1
0
1
DE
T3-vs-T0
T12-vs-T0
both
Coagulation cascade
Complement cascade
Extrinsic pathway
Intrinsic pathway
Alternative pathway
Classical pathway
Lectin pathway
Tissue damage
Contact with damaged vessel
Microbe
Antigen-antibody complex
Carbohydrate
F12
KLKB1
PLG
F3
F11
SERPINC1
KNG1
BDKRB1
Kallikrein-kinin system
F7
F9
Inflammatory mediator regulation of TRP channels
Inflammation, vasodilation
VWF
F8
TFPI
F10
F5
PROS1
Cell adhesion, migration, proliferation
PLAUR
SERPINC1
SERPIND1
SERPINA5
F2
(Thrombin)
THBD
PROC
SERPINE1
PLAU
PLAT
PROCR
SERPINA5
SERPINA1
SERPINF2
A2M
Plasmin
F2R
F2RL2
Anti-inflammatory responses,vasodilation, increased endothelial permeability, release of Weibel-Palade bodies
Platelet activation
F13A1
CPB2
Fibrinolytic system
FGG
Fibrin monomer
Cross-linked fibrin polymer
Fibrin clot
Fibrin degradation products
C3(H2O)
CFH
CFI
C1R
MBL2
MASP2
SERPING1
C3(H2O)Bb (C3 convertase)
CFB
CFD
C2
C4A
C3
C3a
CFI
CD55
CD46
LOC100138386
C2b
C4a
C3bBb (C3 convertase)
C3
C4b2a (C3 convertase)
C4BPA
LOC101905630
LOC519127
CFH
C3a
C3AR1
Degranulation, chemotaxis
Amplification loop
C3b
C3b
VSIG4
Phagocytosis
C3f
LOC100138386
C3bBb3b (C5 convertase)
C5
C4b2a3b (C5 convertase)
iC3b
CR2
B cell receptor signaling pathway
C5a
C3c
ITGB2
Phagocytosis
C5b
C3dg
C8G
ITGB2
C3d
CD59
CLU
VTN
Membrane attack complex (C5b-9)
Cell lysis
C5AR1
Degranulation, chemotaxis
Data on KEGG graph
Rendered by Pathview

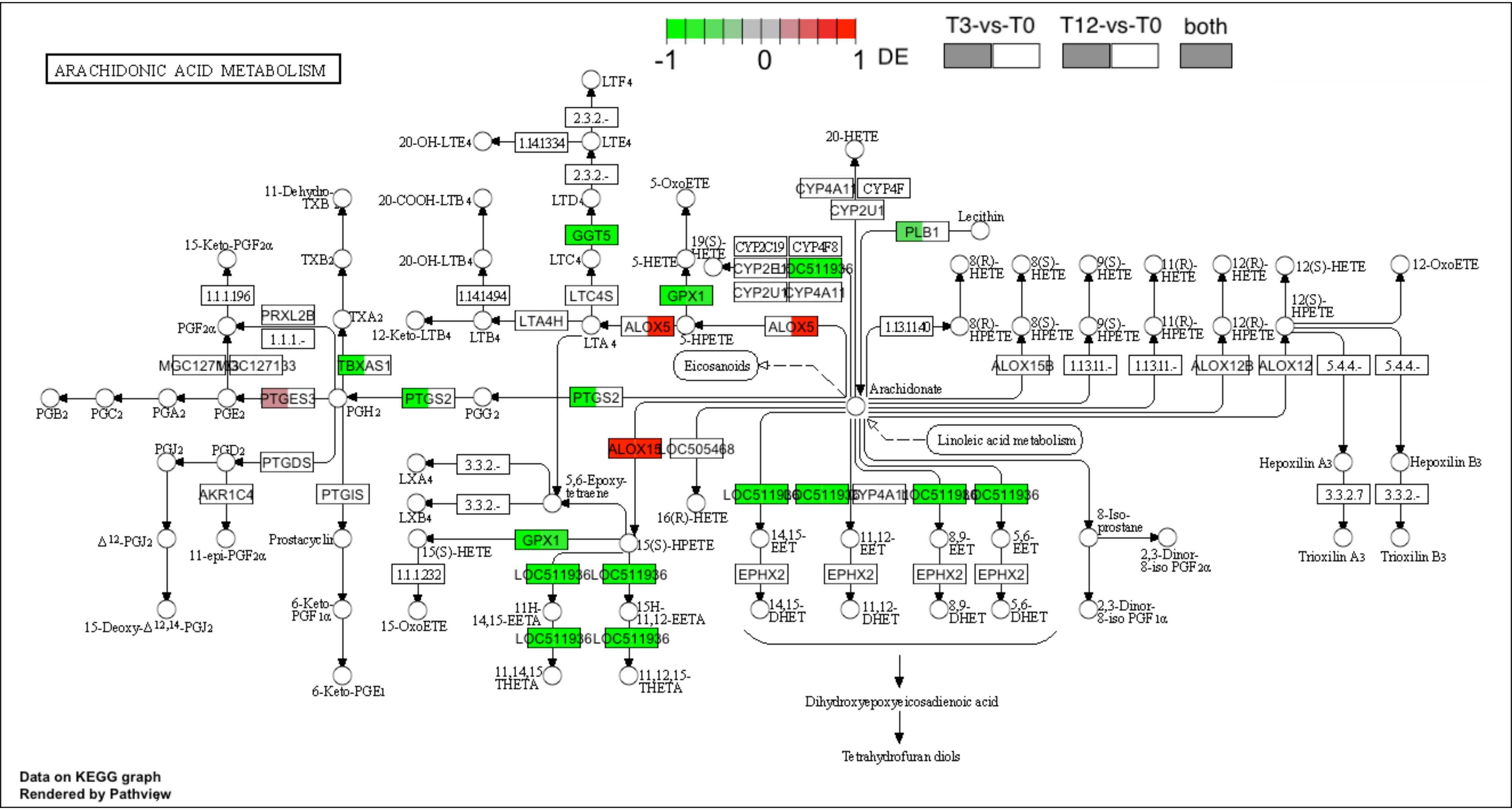
ARACHIDONIC ACID METABOLISM
-1
0
1
DE
T3-vs-T0
T12-vs-T0
both
LTF4
2.3.2.-
20-OH-LTE4
1.14.13.34
LTE4
2.3.2.-
LTD4
GGT5
LTC4
LTC4S
20-COOH-LTB4
20-OH-LTB4
1.14.14.94
12-Keto-LTB4
LTB4
LTA4H
LTA4
11-Dehydro-TXB2
TXB2
TXA2
TBXAS1
15-Keto-PGF2α
1.1.1.196
PGF2α
PRXL2B
1.1.1.-
PTGES3
PGH2
PTGS2
PGG2
PGB2
PGC2
PGA2
PGE2
PGJ2
PGD2
PTGDS
AKR1C4
PTGIS
Δ12-PGJ2
11-epi-PGF2α
Prostacyclin
6-Keto-PGF1α
6-Keto-PGE1
15-Deoxy-Δ12,14-PGJ2
LXA4
LXB4
3.3.2.-
5,6-Epoxy-tetraene
15(S)-HETE
GPX1
15(S)-HPETE
1.1.1.232
15-OxoETE
11H-14,15-EETA
15H-11,12-EETA
11,14,15-THETA
11,12,15-THETA
5-OxoETE
5-HETE
19(S)-HETE
GPX1
ALOX5
5-HPETE
Eicosanoids
ALOX15
16(R)-HETE
20-HETE
CYP4A11
CYP4F
CYP2U1
CYP2C19
CYP4F8
CYP2E1
Lecithin
PLB1
Arachidonate
Linoleic acid metabolism
14,15-EET
11,12-EET
8,9-EET
5,6-EET
EPHX2
14,15-DHET
11,12-DHET
8,9-DHET
5,6-DHET
Dihydroxyepoxyeicosadienoic acid
Tetrahydrofuran diols
8-Iso-prostane
2,3-Dinor-8-iso PGF2α
2,3-Dinor-8-iso PGF1α
1.13.11.40
8(R)-HETE
8(S)-HETE
9(S)-HETE
11(R)-HETE
12(R)-HETE
12(S)-HETE
12-OxoETE
8(R)-HPETE
8(S)-HPETE
9(S)-HPETE
11(R)-HPETE
12(R)-HPETE
12(S)-HPETE
ALOX15B
1.13.11.-
ALOX12B
ALOX12
5.4.4.-
Hepoxilin A3
Hepoxilin B3
3.3.2.7
3.3.2.-
Trioxilin A3
Trioxilin B3
Data on KEGG graph
Rendered by Pathview

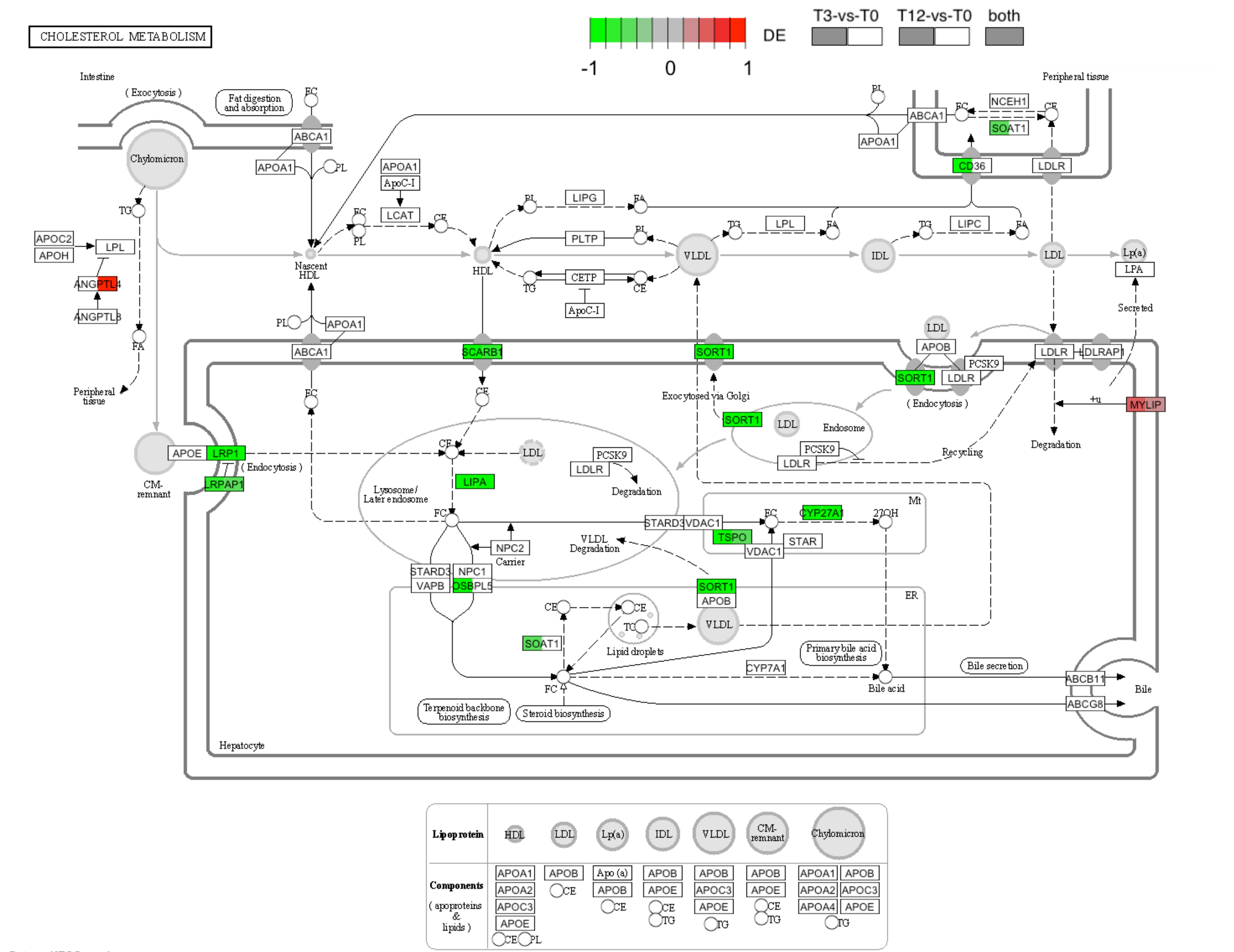

Data on KEGG graph
Rendered by Pathview
